# Microbial community composition in the rhizosphere of *Larix decidua* under different light regimes with additional focus on methane cycling microorganisms

**DOI:** 10.1038/s41598-020-79143-y

**Published:** 2020-12-18

**Authors:** Nadine Praeg, Paul Illmer

**Affiliations:** Department of Microbiology, Universität Innsbruck, Technikerstrasse 25d, 6020 Innsbruck, Austria

**Keywords:** Microbial ecology, Soil microbiology, Archaea, Bacteria, Fungi, Microbiome, Symbiosis, Ecology

## Abstract

Microbial community and diversity in the rhizosphere is strongly influenced by biotic and/or abiotic factors, like root exudates, nutrient availability, edaphon and climate. Here we report on the microbial diversity within the rhizosphere of *Larix decidua*, a dominant tree species in the Alps, as compared with the microbiome within the surrounding soil. We describe how increased light intensity influenced the rhizobiome and put emphasize on methane cycling microorganisms. Microbial taxa were classified into 26 bacterial, 4 archaeal and 6 fungal phyla revealing significant differences between bulk and rhizosphere soils. The dominant prokaryotic phyla were Proteobacteria, Acidobacteria, Actinobacteria (both, rhizosphere and bulk soil) and Bacteroidetes (rhizosphere soil only) and dominant fungal phyla in both fractions included Ascomycota and Basidiomycota. The rhizosphere community was indicated by *Suillus* sp., plant growth-promoting bacteria and *Candidatus* Saccharibacteria. Predicted genes in membrane transport and carbohydrate metabolism were significantly more abundant in rhizosphere soils while genes connected with energy metabolisms and cell motility increased in bulk soils. Dominant methanotrophic microorganisms were Upland Soil Cluster (USC) α methanotrophs, *Methylogaea* spp. and *Methylosinus* spp., while most methanogens belonged to Methanomassiliicoccales. The overall abundance of methanotrophs distinctly increased in the rhizosphere but to a very different species-specific extent. The increased light intensity only led to minor changes in the rhizobiome, nevertheless a couple of indicator species (e.g. *Pseudomonas* sp.) for intensified light conditions were established.

## Introduction

Microbial communities make an important contribution to the metabolic processes performed in soils. About 90% of soil functions, including the decomposition of organic matter, fixing of nitrogen, increasing the bioavailability of nutrients to plants and soil organisms, storing carbon in soil humus and actively or passively releasing carbon dioxide (CO_2_) and methane (CH_4_) to surrounding soil and air, are performed by microorganisms^1, 2^. Bacteria, archaea and fungi constitute the soil microbiome and the compositional (and functional) structure is shaped by climatic and edaphic factors. A biologically very active zone in soils is the rhizosphere. Plants live in association with a great number and diversity of microorganisms, the so-called rhizobiome. Plant–microbe interactions in the rhizosphere can be beneficial to the plant, the microorganisms, to both or to neither of them, but can also be negative (pathogenic). Plants deposit various, often labile root exudates that improve the carbon-limited conditions in soils^3^. Rhizodeposition describes the total carbon (C) flow from the plant roots to the soil. 10–40% of the photosynthetically fixed C is excreted by the roots including organic acids, sugars, amino acids, lipids, coumarins, flavonoids, proteins, enzymes, aliphatics and aromatics all leading to an increased C-turnover^4,5^. Such hotspots show higher microbial activities and abundances and altered community compositions compared to the surrounding bulk soil^3,4,6,7^. Furthermore, the rhizodeposition affects the symbiotic associations between plant and soil microorganisms^5^ and plant-associated microorganisms are known to influence seed germination, plant growth and development, nutrition, diseases and productivity^6^.

In forest ecosystems, soil bacteria and fungi play an essential role in biogeochemical cycles and nutrient transformations^8^ and thus also have important climate functions, actively and passively. As part of the carbon cycle, CH_4_ accounts for a small proportion of the total global C budget, but it is ecologically of great concern. Methane is with a current atmospheric concentration of ~ 1.86 ppm^9^ the second most important atmospheric greenhouse gas after CO_2_ and is believed to account for 20% of global warming^10^ but knowledge about how the belowground C supply by plants’ roots influences the CH_4_ balance is still limited. Most of the atmospheric CH_4_ is produced by methanogenic archaea and most representatives of methanogenic species belong to Euryarchaeota, but it was shown that outside of the Euryarchaeota, the candidate phyla Bathyarchaeota (former Miscellaneous Crenarchaeota Group) and Verstraetearchaeota harbor mcr-like genes that are responsible for methane production^11^. In contrast, aerobic methane-oxidizing bacteria (MOB) are responsible for the biological oxidation of CH_4_ and either use the particulate (pMMO) and/or soluble (sMMO) methane monooxygenase enzymes for oxidizing methane to methanol in the initial step^12^. Based on the central carbon pathway of methanotrophs, orientation and distribution of intracytoplasmatic membranes and composition of lipids in terms of fatty acid proportions^13^, methanotrophs can be categorized into type I methanotrophs (Gammaproteobacteria), type II methanotrophs (Alphaproteobacteria) and type III methanotrophs (Verrucomicrobia)^12–14^. Furthermore, a subset of currently uncultivated methanotrophs are observed in multiple investigations in upland soils^15,16^ and have according to phylogenetic analysis of the pMMO (namely the subunit *pmoA*) been classified into Upland Soil Clusters (USCα and USCy).

Within the present investigation, we aimed to determine the rhizobiome of *Larix decidua* (*L. decidua* Mill, European larch) and compared the microbial composition with the adjacent rootless soil. The influence of the plant on the rhizobiome was varied and modulated by different light intensities under which the tree saplings grew. It was our hypothesis that an increase in light intensity can alter the rate of photosynthesis and thus the root exudates that could affect the rhizobiome in return. Changing light intensities can either occur on daily basis but also along with climate change and alterations in stock density. *L. decidua* was selected as it is the second most common coniferous tree species in Austria and represents a dominant and valuable tree species in the Alps in general, mainly in the subalpine belt of the Central Alps^17,18^. From an ecological point of view, *L. decidua* is characterized by a high adaptability to warmer environmental conditions, especially in comparison to the currently very dominant conifer species *Picea abies* (Norway spruce)^19^ and has thus received increasing attention in the last years. To the best of our knowledge, there is currently no investigation dealing with the characterization of the prokaryotic and fungal rhizobiome composition of *L. decidua*, especially not under the influence of changed light intensities.

In the present study, we hypothesize that a change in light intensity could alter and accelerate the activity of *L. decidua* and thus the rate of photosynthesis which in return could affect the rhizobiome. A further idea behind changing the light conditions was to include the occasion that especially at higher elevations, larch forests do not form closed forest stands but rather loose stands with smaller trees which is frequently found in the Central European Alps. As a result, light intensities on the forest soil are as high as in the canopy^20^. Thus, the aims of the study were to (1) analyze the microbiome in the rhizosphere of *Larix decidua* in comparison to that of the surrounding soil, (2) investigate whether *Larix* specifically affects microbial communities of methanogenic and methanotrophic communities and (3) investigate whether increased light intensities can alter the plant effect on microbial communities.

## Results

### Microbial community composition in bulk and rhizosphere soil: prokaryotes

**Figure 1:**
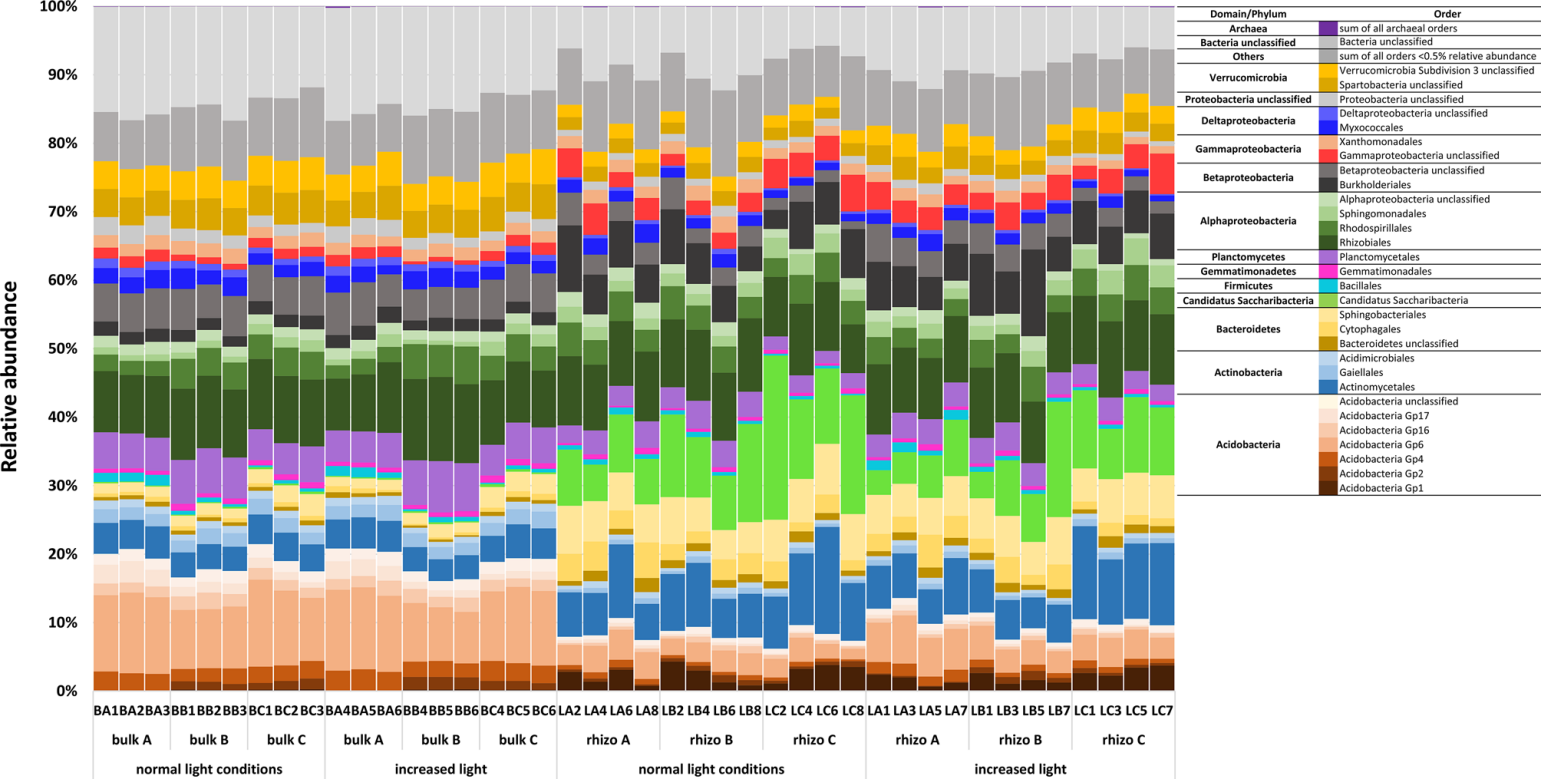
Community composition of prokaryotes on order level representing most abundant orders (95% of relative abundance) in bulk and rhizosphere (rhizo) soils at normal and increased light conditions. A,B,C represent the forest replicate sites. BA, BB, BC stand for bulk soils on site replicate A, B and C. LA, LB, LC stand for rhizosphere soil of *L. decidua* (L) on site replicate A, B and C. Numbers indicate replicates within fraction, site and light treatment.

Prokaryotic community composition was dominated by Bacteria while Archaea made up only ~ 0.1% of the total prokaryotic community. Bacterial communities in the investigated forest bulk and rhizosphere soil samples were composed of 13,492 different operational taxonomic units (OTUs) mainly belonging to the phyla of Proteobacteria (36% on average including all samples), Acidobacteria (16%), Actinobacteria (11%), Bacteroidetes (7%), *Candidatus* Saccharibacteria (6%), Verrucomicrobia (5%) and Planctomycetes (4%). Figure 1 shows the most abundant prokaryotic orders (> 95% of relative abundance) in bulk and rhizosphere soil samples. Regarding Bacteria, the greatest difference between bulk and rhizosphere soil was reflected in the significantly higher abundance of *Candidatus* Saccharibacteria in the rhizosphere fraction in which they constituted up to 10% on average of the total relative abundances compared to about 0.3% in bulk soils. This candidate phylum was present with > 120 different OTUs in the rhizosphere while only a few occurred in bulk soils. Furthermore, the phylum Bacteroidetes was significantly more abundant in rhizosphere soils. In return, the relative abundance of Acidobacteria, Planctomycetes, Verrucomicrobia and unclassified Bacteria decreased in the rhizosphere fraction while the phylum of Proteobacteria on phylum level did not significantly differ between the fractions. Detailed information on the existing taxa (on genus level) and significant differences between the fractions are shown in Fig. S1 and Table S1 (see Supplementary Information), respectively. Community differences according to the replicate sites used in this study, e.g. changes within the acidobacterial groups, were established and may also be linked to minor changes in soil properties (i.e. ammonium concentration). Sphaerobacterales, Caldilineales and Bacillales increased on site A while the acidobacterial groups 1, 2 and 3 significantly decreased on this site. Basic soil properties are presented in Table S2 (see Supplementary Information). The most pronounced changes in the soil properties on site A were reflected in decreased ammonium concentration and slightly increased pH.

The archaeal community composition was dominated by the phylum Thaumarchaeota (65% of total archaeal abundances on average). A proportion of 22% of total archaeal abundances stayed unclassified on Euryarchaeota level and was distinctly lower within the rhizosphere (25.9% and 18.8% in bulk and rhizosphere soil, respectively). Besides the amount of unclassified Euryarchaeota, 3.8% of the classified Euryarchaeota were identified as methanogens. Pacearchaeota and Woesarchaeota completed the composition of the archaeal community with 2% relative abundance each and showed no significant differences between bulk and rhizosphere samples. On phylum level, the influence of increased light intensity did not lead to compositional differences in the prokaryotic community structure, but higher taxonomy levels (class level onwards) highlighted significant influences of additional light on the rhizobiome (please see “Identification of biomarkers” section). Bulk soil prokaryotic communities were not influenced by additional light.

**Figure 2:**
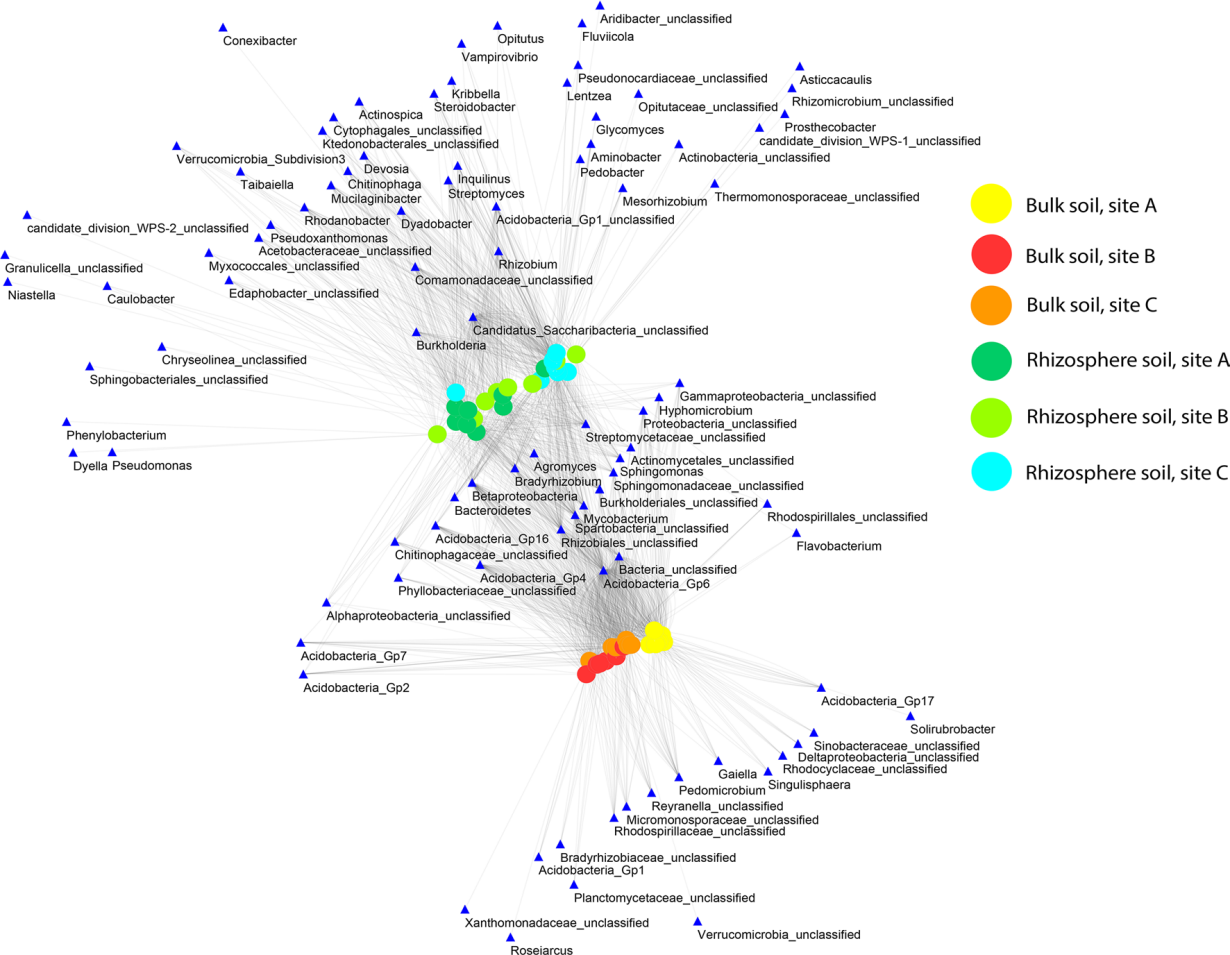
Network analysis of OTUs (97% similarity) shared between bulk and rhizosphere soils of all replicate sites and both light approaches. Soil samples are represented as colored circles while OTUs are represented as blue triangles. The classification of each group is written above the node. *OTU*, operational taxonomic unit.

The microbiome of the soil itself and the rhizobiome of *L. decidua* had numerous OTUs in common but distinctly expressed indicators for the one or the other fraction (Fig. 2). The visualized bipartite network shows that all OTUs divided into three clusters (Fig. 2). One cluster emerged with bulk soils, another with soil samples from the rhizosphere soils and a third, connecting cluster of OTUs and groups between those two. Thus, the network highlights taxa that occurred in both fractions and taxa that existed only in one or the other faction. Highly connecting groups between bulk and rhizosphere clusters included representatives of Acidobacteria Gp4, 6 and 16, Rhizobiales, Spartobacteria, *Bradyrhizobium* sp. and *Mycobacterium* sp. Correlations between the relative abundances of prokaryotic phyla are presented in Fig. S2 (Supplementary Information) and highlight shared in- or decreases of prokaryotic classes in bulk and rhizosphere soils. Significant correlations between the relative abundances of several prokaryotic phyla were established and distinctly more (positive) relationships were found in rhizosphere soils compared to bulk soils.

### Microbial community composition in bulk and rhizosphere soil: fungi

**Figure 3:**
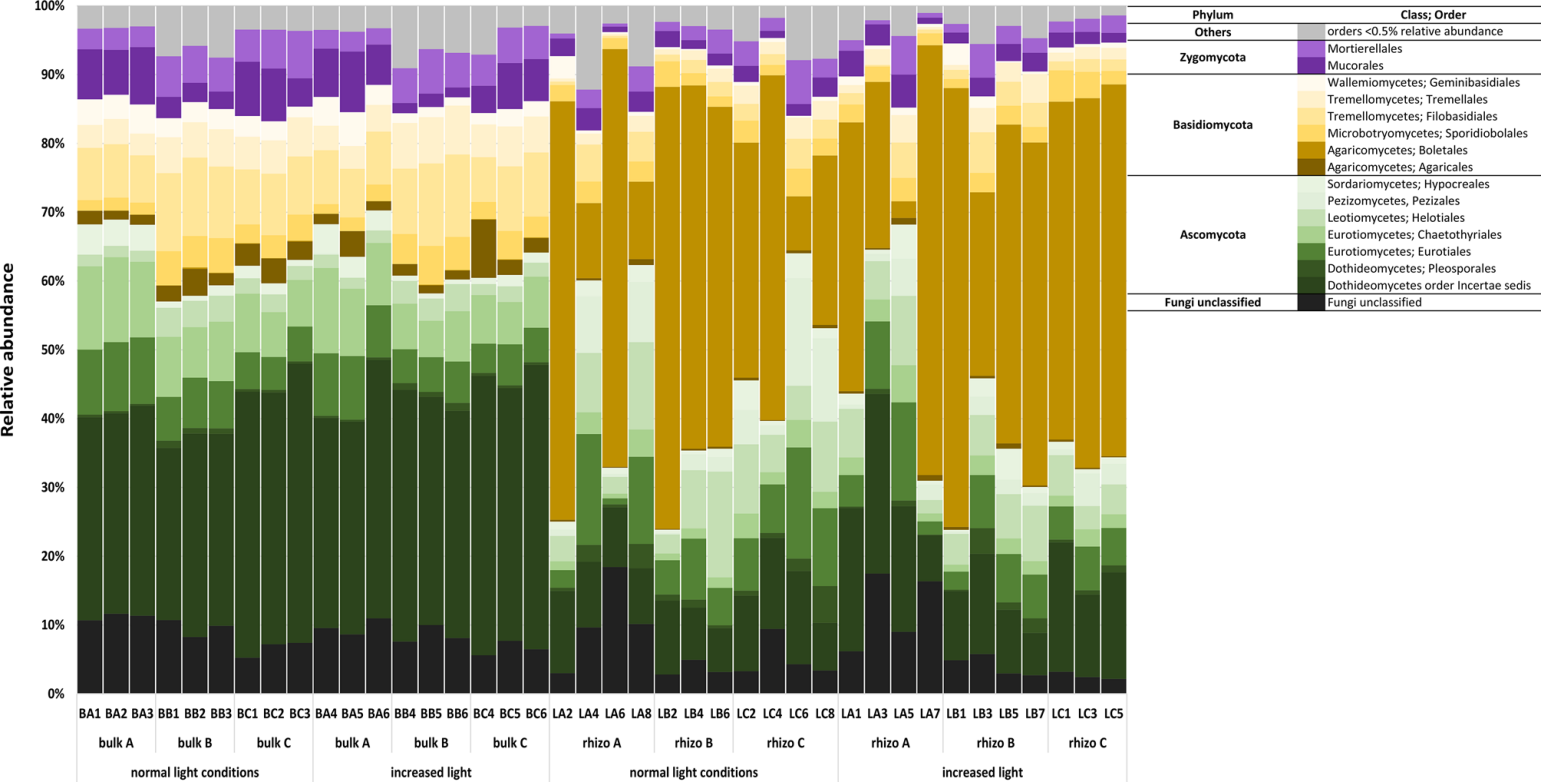
Community composition of fungi on order level representing most abundant orders (95% of relative abundance) in bulk and rhizosphere (rhizo) soils at normal and increased light conditions. A,B,C represent the forest replicate sites. BA, BB, BC stand for bulk soils on site replicate A, B and C. LA, LB, LC stand for rhizosphere soil of *L. decidua* (L) on site replicate A, B and C. Numbers indicate replicates within fraction, site and light treatment. LB8 and LC7 were excluded for the description of the fungal community composition as the samples did not meet the quality requirements.

Soil fungal communities included 4092 OTUs and were primarily composed of Ascomycota (47% on average of all ITS reads), Basidiomycota (38%) and Zygomycota (7%). In more detail, Ascomycota were present with 56.5% in bulk soils, 38.9% in rhizosphere soils and Basidiomycota with 23.9% in bulk soils and 49.0% in rhizosphere soils. The most dominant Ascomycetes orders were Dothideomycetes (33.5% in bulk soils, 15.0% in rhizosphere soils) and Eurotiomycetes (15.0% in bulk soils, 10.1% in rhizosphere soils). Further abundant classes within Ascomycota were Leotiomycetes, Sordariomycetes and Pezizomycetes. Basidiomycetes were dominated by Agaricomycetes with 39.7% vs. 3.7% in rhizosphere and bulk soils, respectively. Further classes representing Basidiomycota were Tremellomycetes, Microbotryomycetes and Wallemiomycetes and Mortierellales and Mucorales were the two most dominant orders of Zygomycota in both fractions. The biggest difference between the soil fractions was the significant higher abundance of *Suillus* (*grevillei*) (Agaricomycetes) in the rhizosphere. Figure 3 shows the most abundant orders in bulk and rhizosphere soil samples. Comparable to prokaryotes, differences in fungal taxa depending on the replicate sites studied were also established and especially applied for site A, e.g. Sebacinales was present on site A only. Detailed information on the occurring taxa (on genus level) and significant differences in the fractions are shown in Fig. S3 and Table S1 (see Supplementary Information), respectively.

### Alpha- and beta diversity of prokaryotic and fungal communities in bulk and rhizosphere soil of *L. decidua*

**Figure 4:**
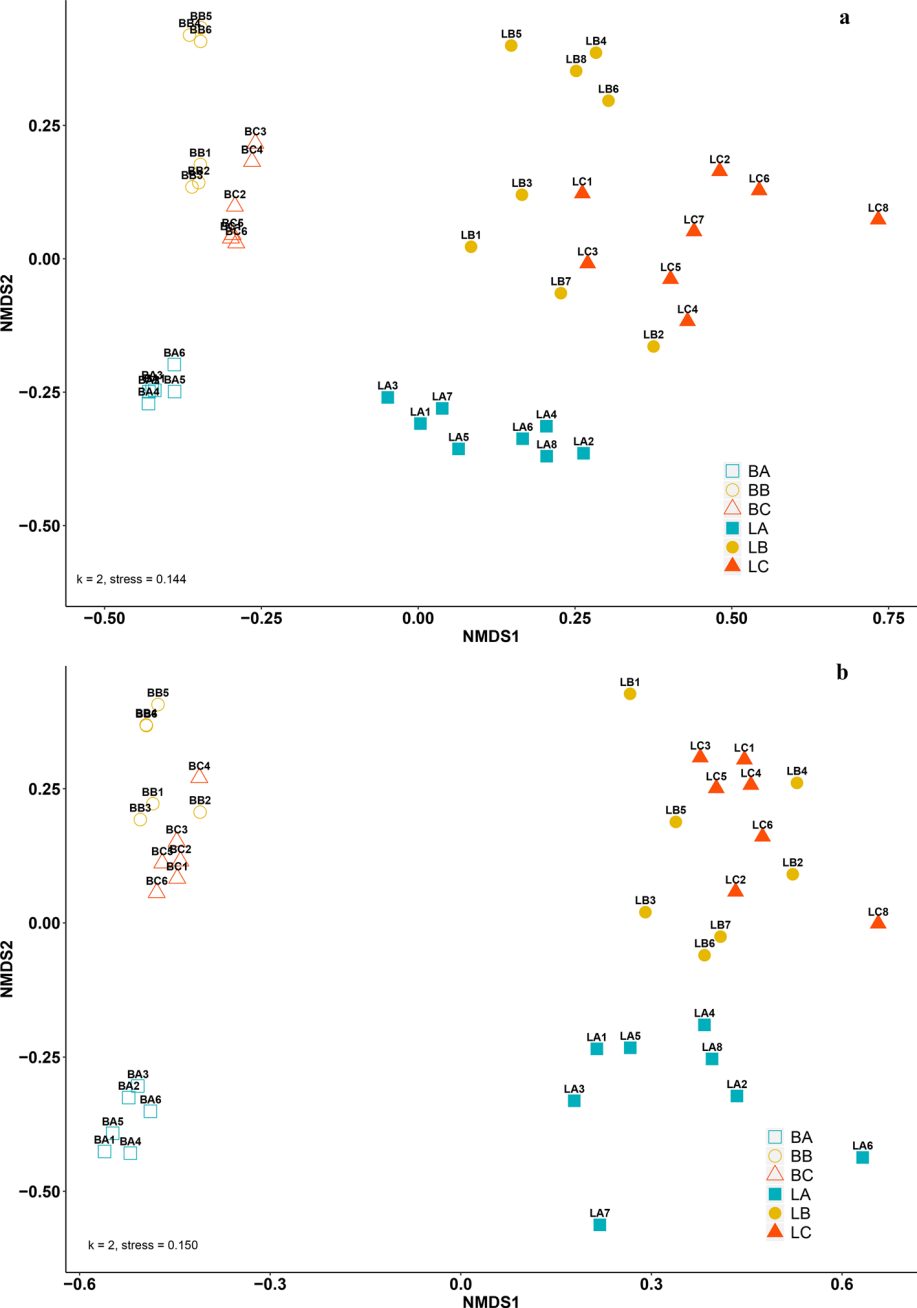
Non-metric multidimensional scaling (NMDS) plot displaying β-diversity by Bray–Curtis dissimilarities for (**a**) prokaryotic and (**b**) fungal communities. Non-filled symbols show bulk soils (B), filled symbols show rhizosphere samples of *L. decidua* (L). Letters (A, B, C) indicate the site replicates and numbers represent the respective replicates within fraction, site and light treatment (e.g. BA1 stands for the community in bulk soils at forest site A and sample replicate 1). Numbers in bulk soils 1, 2, 3 stand for normal light and 4, 5, 6 for intensified light conditions. Numbers in rhizosphere samples 1, 3, 5, 7 indicate intensified light and 2, 4, 6, 8 normal light conditions.

α-diversity including the diversity index (Shannon) and richness index (Chao) for prokaryotic and fungal communities in bulk and rhizosphere soil are shown in Fig. S4 (see Supplementary Information). Diversity of prokaryotes was significantly influenced by fraction (bulk vs. rhizosphere). No significant influence was ascertained for the different light treatments. Prokaryotic richness (Fig. S4b) was influenced by the different replicate sites and was increased in rhizosphere soils compared to bulk soils in case of replicate A and B, and decreased at site C. Fungal diversity was quite homogeneous and condensed in bulk soils while rhizosphere soils showed varying diversities (Fig. S4c). Fungal richness was significantly increased in the rhizosphere compared with the bulk soil samples (Fig. S4d). Including the total OTU matrix, the rhizosphere of *L. decidua* significantly influenced the prokaryotic and fungal community composition of the studied forest soil. Figure 4 shows a summary of the β-diversity of the prokaryotic (a) and fungal (b) community including all replicates and different sites. The NMDS analysis of the prokaryotic communities revealed that the two fractions (bulk and rhizosphere) formed own clusters separated by the first axis. In both, fungal and prokaryotic cases the replicate sites of the bulk soils clustered quite close to each other while samples within the rhizosphere differed more distinctly. For the prokaryotic and fungal distance matrix, the influence of fraction (bulk vs. rhizosphere) on total OTU matrix was significant (Prokaryotes: Bray–Curtis R_ANOSIM_ = 0.948, p < 0.001, Fungi: Bray–Curtis R_ANOSIM_ = 0.8014, p < 0.001). In the case of prokaryotes, the influence of increasing light on the community composition in the rhizosphere was only moderate whereas the influence of the site replicates was weak but anyway significant (Bray–Curtis R_ANOSIM_ = 0.245, p < 0.01).

### Identification of biomarkers

**Figure 5:**
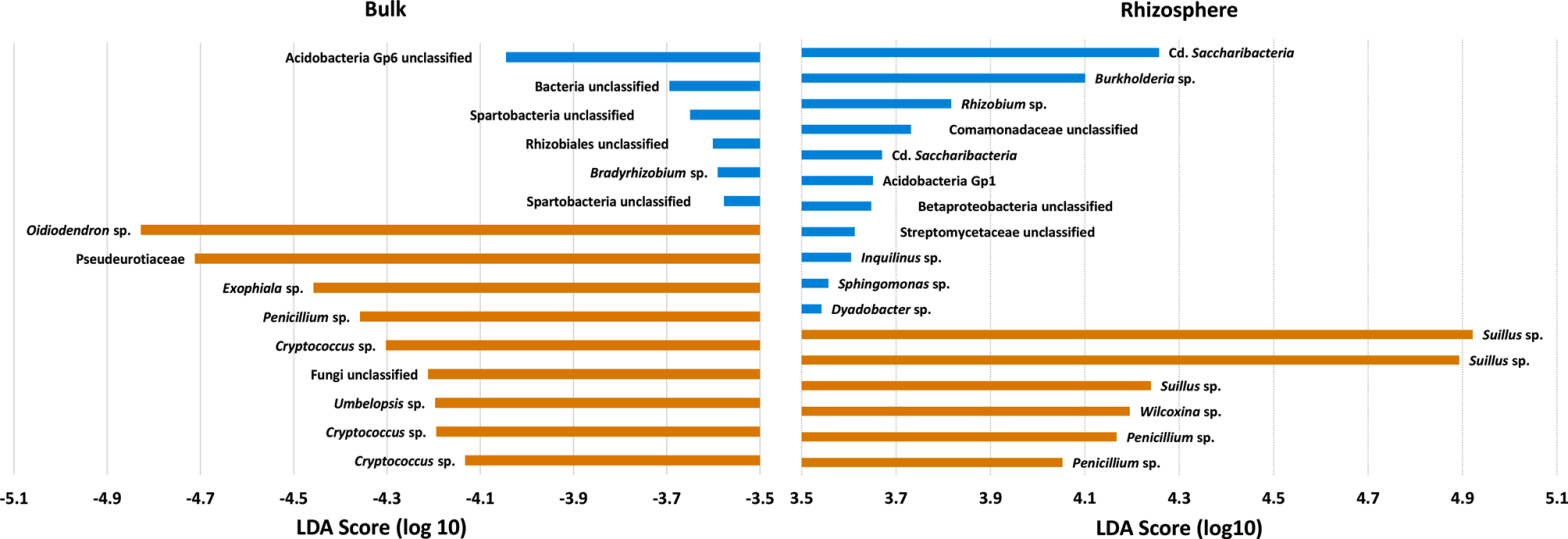
Linear discriminant analysis Effect Size (LEfSe) (log_10_ LDA score) of prokaryotic (blue) and fungal (orange) OTUs detected as indicator species for bulk (left) and rhizosphere (right) of *L. decidua*. Prokaryotic and fungal OTUs are classified at the highest resolvable tax level.

The eight orders Burkholderiales, Actinomycetales, Sphingobacetriales, Cytophagales, Xanthomonadales, Sphingomonadales and Acidobacteria Gp1 were highly significant indicator orders for the rhizosphere fraction. On the contrary, Acidobacteria Gp 4, 6, 16, 17, Planctomycetales, Gaiellales and Spartobacteria highlighted the bulk soils. Orders are given in descending importance. In all, the Linear discriminant analysis Effect Size (LefSe) detected 80 prokaryotic OTUs that highly (LDA score > 3) contributed to the significant difference in the community composition between bulk and rhizosphere soil. In case of fungi, 54 biomarkers were detected via LEfSe with most of them belonging to the phylum Ascomycota but the most pronounced differences were apparent with *Suillaceae* (Basidiomycota). On order level, bulk soil samples were indicated by the orders Agaricales, Geminibasidiales, Mucorales, Tremellales, Chaetothyriales, Filobasidiales and representatives of Dothideomycetes order incertae sedis. The most distinct microbial biomarkers on feature level for bulk and rhizosphere soils are shown in Fig. 5.

**Figure 6:**
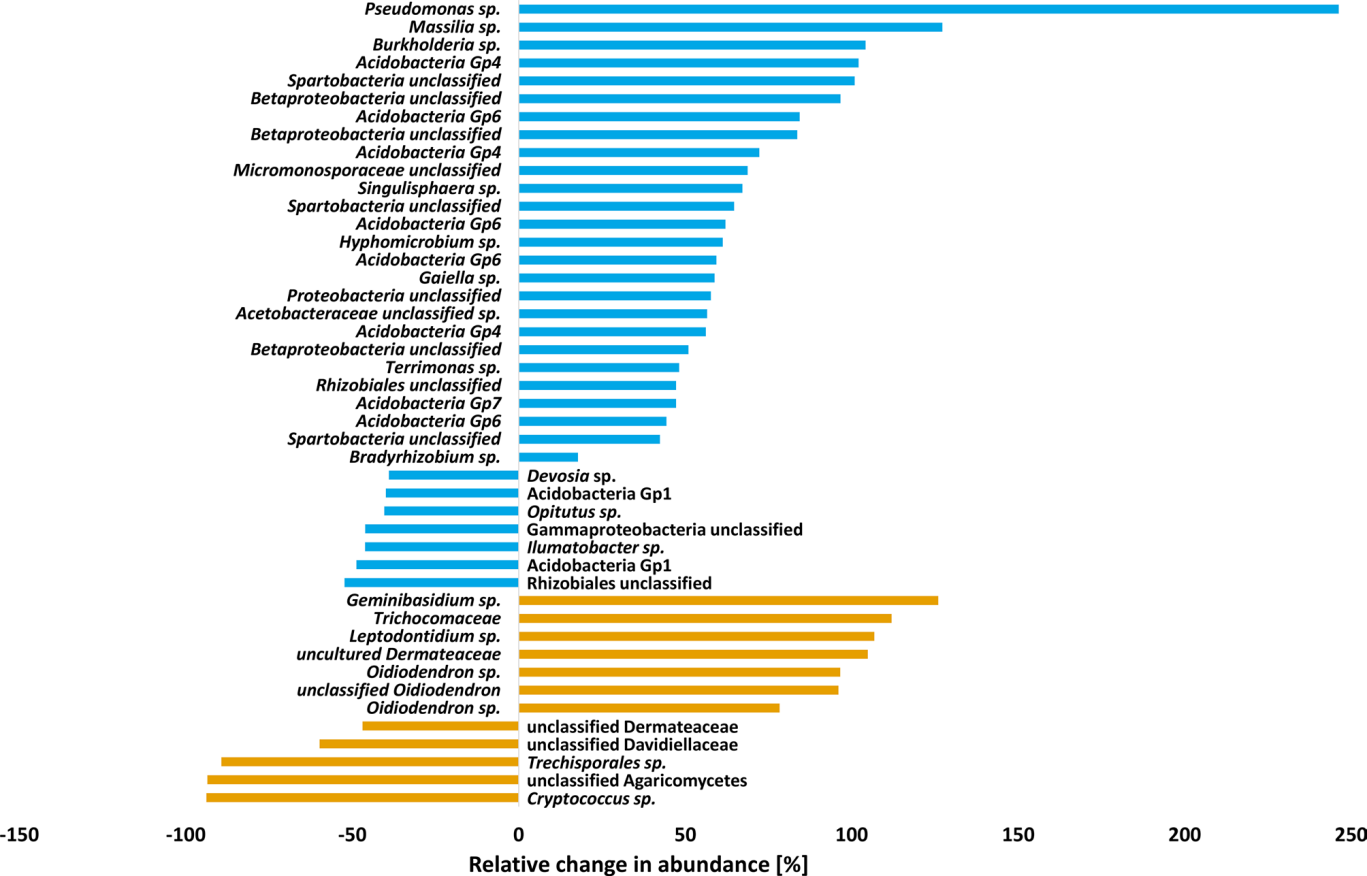
Relative change in abundance [%] of bacterial and fungal OTUs in the rhizobiome that increased (right) or decreased (left) due to the additional light treatment compared to normal light conditions. OTUs are named according to the highest resolved tax level. Blue: bacteria, orange: fungi.

The influence of light on the prokaryotic and fungal community composition in the rhizosphere led to an in- or decrease of several OTUs. A highly significant increase in the relative abundance due to increased light treatment was reached for *Pseudomonas* sp. (Fig. 6). Further increased relative abundances could be assigned to *Massilia* sp., *Burkholderia* sp. and several OTUs classified as Acidobacteria while other groups within Acidobacteria in return showed decreased abundances due to the increased light treatment. Thus, the bacterial taxa Spartobacteria, Pseudomonadaceae, Acidobacteria Gp4 and Gp6 were the taxa mostly affected by the light regime. Fungal biomarkers for the light variants were identified as *Geminibasidium* sp. and three *Oidiodendron* species for additional light while *Cryptococcus* sp. decreased under additional light conditions (Fig. 6). Thus, the most affected families due to the intensified light regime were Geminibasidiaceae, Trichocomaceae and Dermataceae.

### Functional predictions and metagenomic biomarkers

**Figure 7:**
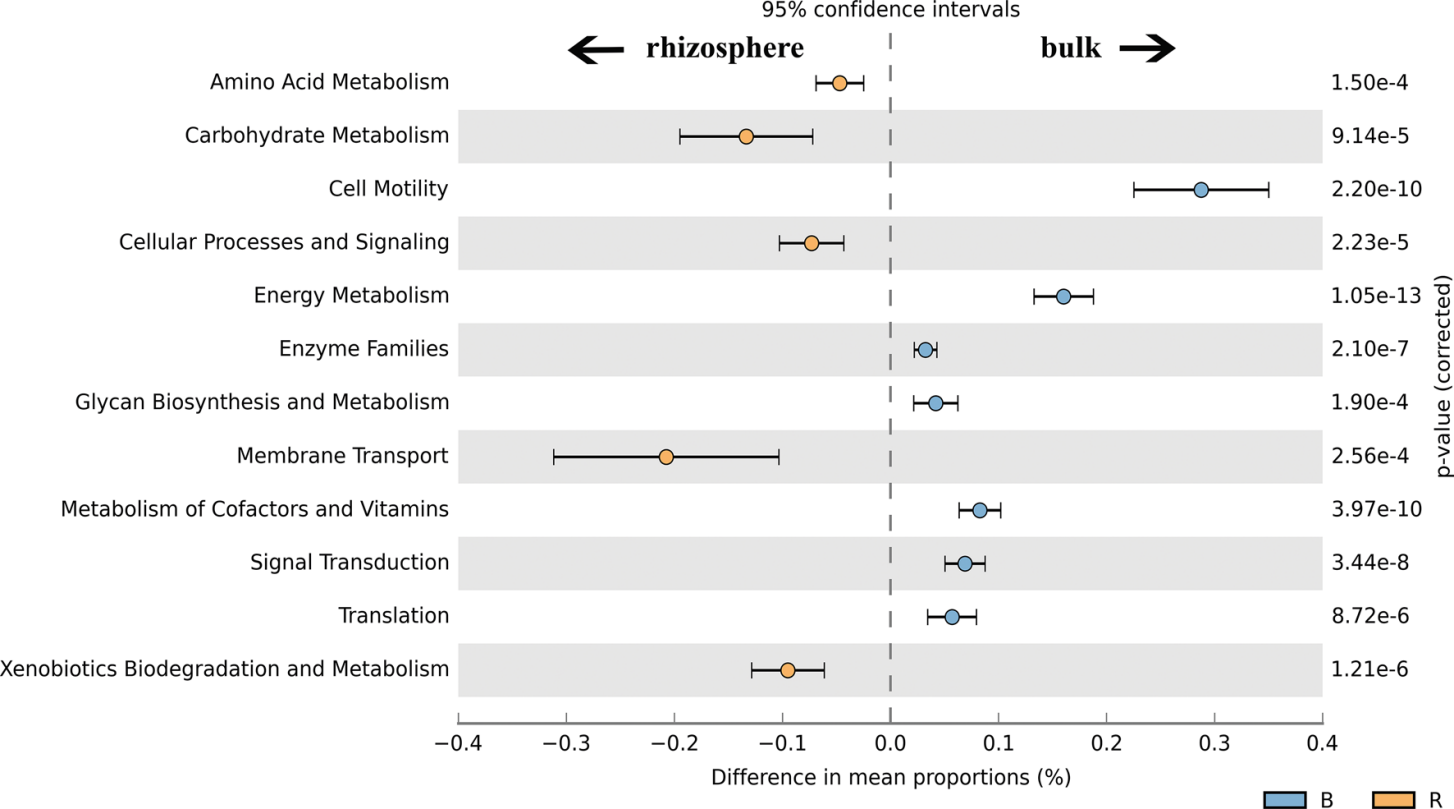
Extended error bar plot indicating the difference in mean proportion [%] of the predicted gene abundance in bulk (B, blue) and rhizosphere (R, orange) soil. p-values were corrected by using Bonferroni adjustment for multiple test correction.

For understanding the metabolic potential of the investigated soil and identifying differentially abundant functional traits, metagenomes were predicted by PICRUST using 16S rRNA amplicons. The predicted functions were classified as KEGG Orthologs (KOs) resulting in 6909 KOs across all samples. The KOs were further categorized by function on a KEGG Pathway Hierarchy Level of 3. For a variety of functional predictions significant differences could be established (Fig. 7). Predicted genes in membrane transport and carbohydrate metabolism were significantly more abundant in rhizosphere soil samples while energy metabolisms and cell motility were more abundant in bulk soils (Fig. 7). Regarding membrane transport, genes responsible for the increased abundance in the rhizosphere included many ATP-binding proteins responsible for (sugar) transport through the membrane. These functions are highly energy-demanding and are involved in high-affinity uptake of small molecules. In case of carbohydrate metabolism, predicted enzymes included especially enzymes involved in the pentose-phosphate pathway, pentose and glucoronate interconversions, fructose and mannose metabolism and glyoxylate and dicarboxylate metabolism. In bulk soil samples, genes included in nitrogen metabolism, carbon fixation pathways, oxidative phosphorylation, sulfur and methane metabolism were predicted to be more abundant and thus, an increased predicted gene abundance regarding the category energy metabolism was established. Predicted cell motility genes included flagellar biosynthesis proteins, proteins responsible for flagellar assembly and chemotaxis proteins and were found to be more abundant in bulk soil samples (Fig. 7).

### Methanogenic and methanotrophic community composition in bulk and rhizosphere soil

**Figure 8:**
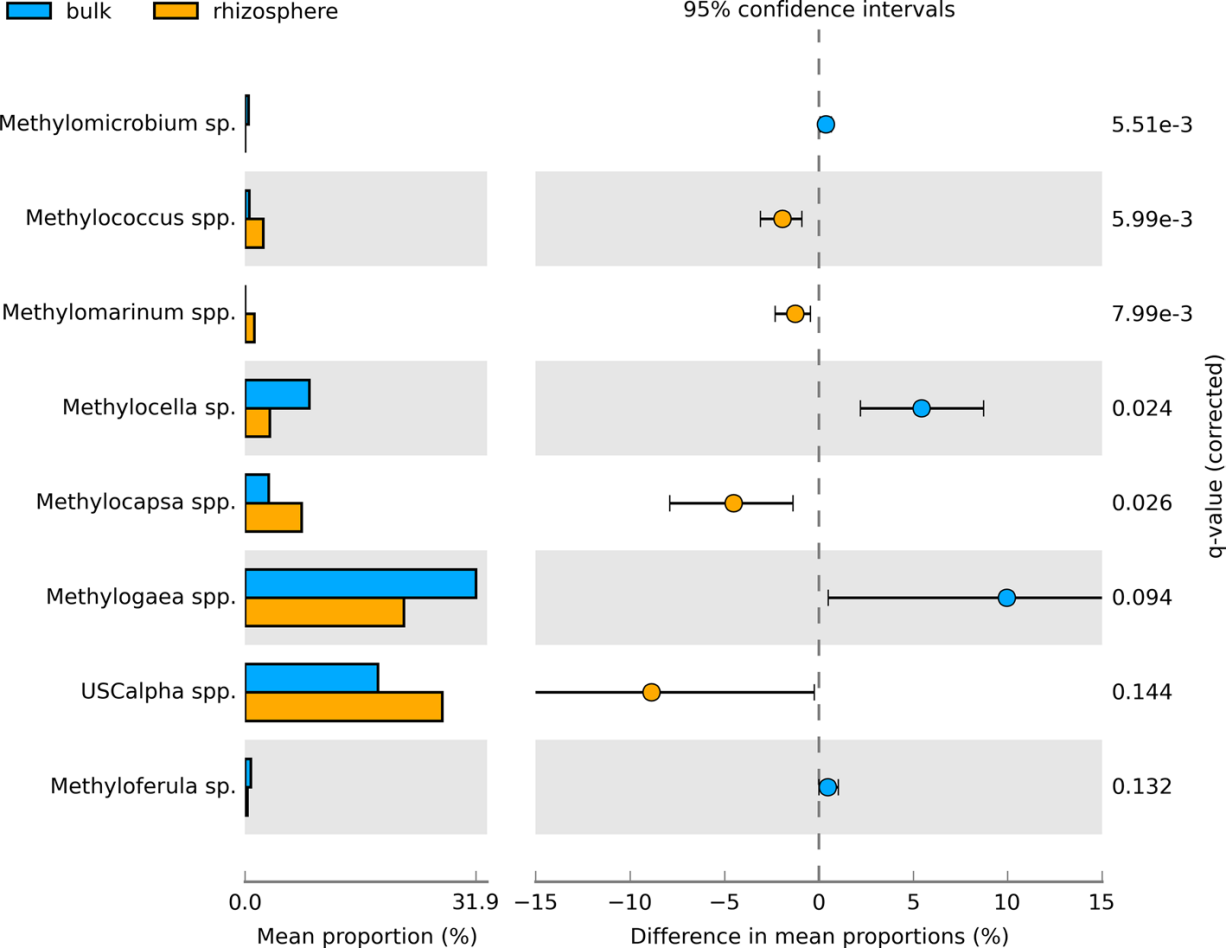
Extended error bar plot showing mean proportions [%] of methanotrophs and indicating differences in mean proportions [%] related to bulk soil (blue) and rhizosphere soil (orange). q-values are adjusted p-values following Benjamini–Hochberg method for multiple test correction. Features with confidence intervals on the positive or negative side only are shown which mainly corresponds to a significance level of p ≤ 0.05.

Several members of methane-producing Archaea were present in bulk and rhizosphere soil samples, belonging to the orders of Methanomassiliicoccales (0.9%), Methanococcales (0.7%), Methanomicrobiales (0.7%), Methanosarcinales (0.3%) and Methanocellales (0.3%). Due to the very heterogeneous distribution of methanogenic species, there was no significant difference in these clades between bulk and rhizosphere soil fractions. Of all reads, MOBs were selected according to taxonomic classification during sequence processing and, if necessary, to verification via BLAST and phylogenetic analysis and are shown in Fig. S7 (Supplementary Information). Sequence analysis regarding MOBs revealed that several methane-oxidizing bacteria were present, both in bulk soils as well as in the rhizosphere, and that they were differently affected by the plants and/or additional light. Overall, the methanotrophic community was dominated by three methanotrophic families, namely Methylocystaceae, Beijerinckiaceae and Methylococcaceae. First, Methylocystaceae were represented by OTUs belonging to *Methylosinus* spp., a methanotrophic genus found ubiquitously in soils^15^, with the OTUs, Otu00513 and Otu00968, outlining a 3-times higher relative abundance in the rhizosphere than in bulk soil (Fig. S7). In contrast, *Methylosinus* sp. (Otu00671) significantly increased in bulk soil samples (Fig. S7) and, thus, when amalgamating all *Methylosinus* spp. no significant difference in the relative abundance between the two fractions was established anymore. Second and in addition to the genus *Methylosinus*, characteristic species for forest soils, e.g. *Methylocella* sp. and *Methylocapsa* sp. were present as well and even constituted 20 to more than 40% to the methanotrophic community (Fig. S7). *Methylocella* spp. include species that are well known from forest soils^12^ and were reduced in their relative abundance in the rhizosphere compared with bulk soil in this study (Fig. 8). Furthermore, *Methylocella* spp. were distinctly less abundant in soil A of the three replicate soil sites in the forest (Fig. S7) which might be traced back to the significantly lower amount of ammonium on this respective field replicate. Further methanotrophic OTUs detected in the present forest soil were identified to belong to the genus *Methylocapsa* which belongs to type II methanotrophs and harbors representatives that are aerobic, slightly acidophilic and were also isolated from subarctic permafrost ecosystems^21^. The Otu04528 showed a high proximity to the clade of *Methyloferula* spp. and all three genera (*Methylocella*, *Methylocapsa* and *Methyloferula*) belong to the family of Beijerinckiaceae (Rhizobiales) and to type II methanotrophs, respectively. On family level, groups of further unclassified Rhizobiales were detected that potentially contain methanotrophic species and highlighted an increased abundance in the rhizosphere. To clarify, whether methanotrophs are included in the unclassified Rhizobiales species, the respective sequences were re-analyzed in a maximum likelihood tree (Fig. S5, Supplementary Information). One very abundant OTU (Otu00310) was identified as to be methanotrophic as the sequence very likely matches the upland soil cluster α (USCα) (Fig. S5) with a slightly increased abundance in the rhizosphere (Fig. 8), especially under increased light conditions (Fig. S7). Third and besides the methanotrophic Rhizobiales species, the order Methylococcales constituted the methanotrophic community. Within the order Methylococcales, *Methylogaea* was the dominant genus and showed a strong dependency on the respective field replicates in the forest soil, resulting in an increased relative abundance in the field sites A (Fig. S7). This effect was slightly masked by the influence of the rhizosphere (Fig. S7). Besides, *Methylococcus* spp. were mainly present in the rhizosphere (Fig. 8, Fig. S7). However, although some taxa distinctly decreased in the rhizosphere when compared with bulk soil, others increased and finally contributed to a slight increase of the sum of methanotrophs in the rhizosphere which was confirmed by MANOVA analysis that outlined significant increases of methanotrophs in the rhizosphere but no increase under intensified light conditions was established. Detailed dependencies of the respective methanotrophs to fraction (bulk vs. rhizosphere soil) and normal/intensified light conditions are shown in Figs. 8 and S6, S7 (see Supplementary Information).

## Discussion

Many environmental factors shape the microbial community composition and soil microbial diversity. In this study, the influence of the rhizosphere of *Larix decidua* on prokaryotic and fungal community composition was investigated in a forest soil and we tried to strengthen the possible plant induced effects by the application of additional light. The rhizosphere is a biologically active zone of the soil around plant roots and contains soil-borne microorganisms. Interactions between plants and microorganisms can be beneficial to the plant, to the microorganisms, to neither or both of them, but also be harmful. The studied forest site is dominated by *L. decidua* and represents a forest in the montane belt in the Central Alps. Rhizosphere microbial communities tend to be tree species-specific^22–24^, and thus this study focused on a comprehensive analysis of one single tree species (*L. decidua*) on its rhizobiome as compared to root-free bulk soil also taking the spatial heterogeneity into account. As a varying parameter, the influence of additional light was tested, thus an indirect way to show the influence of increased photosynthesis of the tree seedlings. We tested normal light conditions in the greenhouse and increased light conditions during a 16 h photoperiod simulating sunny, cloudless conditions. Due to the climatic relevance of forest soils as CH_4_ sinks, further attention was paid on microorganisms engaged in CH_4_ cycle.

The prokaryotic and fungal community composition of the rhizobiome was significantly different compared with that of bulk soils. Overall, the dominant bacterial phyla (Proteobacteria, Acidobacteria and Actinobacteria) identified in this study match with other studies that found those taxa to be dominant in forest soils^8,25,26^. Besides these common soil representatives, the taxa Verrucomicrobia, Bacteroidetes and Planctomycetes completed the prokaryotic community structure. In general, forest soils are recognized as spatially heterogeneous ecosystems, which is especially true when comparing forest soils to agricultural or grassland soils. The heterogeneity applies at various scales, ranging from the landscape to micrometer-sized soil pores and aggregates, thus, affecting microbial activity, community composition and activity^27^. In this study, the heterogeneity was reflected in central soil properties that slightly differed between the respective replicate sites in the field, e.g. ammonium concentration was significantly decreased in soil A while pH was lowest in soil C. Nevertheless, the influence of the rhizosphere on the community structure was much stronger and outcompeted the spatial heterogeneity. Regarding heterotrophic species, the most significant change in the rhizosphere was the occurrence of *Candidatus* (*Cd.*) Saccharibacteria, formerly known as Candidate Division TM7, being present in the rhizosphere only. Currently, there is still limited knowledge about the function of this candidate. 16S rRNA gene sequences reported its abundance in soils, sediments, wastewater and animals as well as in clinical environments^28^. Latest results suggested that *Cd.* Saccharibacteria utilize plant-derived carbon as they lack many biosynthetic pathways, among others the 6-phosphofructokinase enzyme being an essential enzyme in the glycolysis pathway^29^. However, its lack is compensated by genes in the pentose-phosphate pathway that is common in case of a specialization to plant sugars. Indeed, we could show that genes of the respective enzymes of pentose-phosphate pathway were more abundant within the rhizosphere. Besides, it was shown that rhizospheric Saccharibacteria encoded cellulosomes for the degradation of plant or microbially derived cellulose and incorporate nucleotides from bacteria that live off plant exudates^29^. The second most distinct difference compared to bulk soil is the significantly higher relative abundance of Bacteroidetes (mainly due to the significant increase of *Dyadobacter* sp., *Chitinophaga* sp. and *Mucilaginibacter* sp.) in the rhizosphere. Thus, certain bacterial taxa tended to be strongly associated with the rhizosphere fraction which is emphasized in the bipartite network. The network analysis furthermore points out the taxa shared by both fractions and which taxa are of central importance in one or the other fraction or for both (e.g. *Cd.* Saccharibacteria for rhizosphere soils, Acidobacteria Gp6 for the shared fraction). Furthermore and equally important, the relative abundance of Gammaproteobacteria, Actinobacteria (*Streptomyces* sp., *Mycobacterium* sp.) and Acidobacteria Gp1 increased in the rhizosphere. An increase of Sphingobacteria in the rhizosphere was also reported in case of Norway spruce and beech^26^. In contrast, increasingly abundant in bulk soils were Acidobacteria Gp4, 6 and 16, Alphaproteobacteria, Betaproteobacteria and Spartobacteria. The latter are the most dominant Verrucomicrobia found in soils and represent a phylum that has a high diversity with members possessing broad ranges of metabolic capabilities^30^. Currently, the class Spartobacteria contains only one sequenced isolate, namely *Chthoniobacter flavus* which is a slow-growing aerobic heterotroph^30^. Besides, rhizobiome analysis of *L. decidua* showed that the proportion and diversity of Acidobacteria was higher in bulk soil as compared to the rhizosphere confirming previous studies^31,32^. According to Fierer et al.^32^, Acidobacteria negatively correlate with carbon pool and thus are known to be less abundant in rhizosphere soils due to the increased C-availability. Comparable little knowledge exists on archaeal rhizospheric communities and in this study, archaea were mainly represented by members within the ammonia-oxidizing genus *Nitrososphaera* and the relative abundance of this genus was significantly higher in the rhizosphere.

Plants influence the abundance and composition of the bacterial community near the root by releasing a range of exudates to the soil, providing nutrients to the microorganisms^33^. The rhizobiome led to the occurrence of several known plant-growth promoting bacteria, like *Rhizobium* spp., and overall 16 features on phylum level were significantly differently abundant in bulk and rhizosphere soils. Besides, many taxa are shared across all the samples and thus define a core microbiome. In the studied forest soil, the shared taxa identified were dominated by several unclassified Betaproteobacteriales and Rhizobiales. Further part of the core microbiome were Bacteroidetes (especially Chitinophagaceae and Sphingomonadaceae), Rhizobiales, Rhodospirillales, Acidobacteria (Gp6) and Spartobacteria. This matches the findings of Fierer et al.^34^ who showed that Proteobacteria, Acidobacteria and Verrucomicrobia tend to dominate the (core) microbial community in temperate and boreal forests. Differences within fungi mainly derived from a significant increase of Basidiomycetes (mainly Suillaceae) in the rhizosphere at the expense of Ascomycetes and Zygomycetes. The increased occurrence of *Suillus* sp. (Boletales) confirms the close symbiotic relationship between *Suillus* (*grevillei*) as an ectomycorrhizal fungi and *Larix decidua*^35^. Comparable to the prokaryotic community, differences for fungal taxa depending on the replicate site in the forest were also established.

Besides analyzing the total OTU matrix, diversity and richness indices were investigated as well. With regard to prokaryotes, the Shannon index and the Chao richness estimator revealed a distinct decreased diversity and richness in the rhizosphere, respectively. Although it is often reported that microbial diversities increase in the rhizosphere and accordingly with increased nutrient availability^36,37^, our results approved formerly proposed hypothesis that habitats tend to have a greater diversity under nutrient-limited conditions^38^. The Shannon index for fungi did not differ between the two fractions but contrary to prokaryotes, Chao as a richness estimator showed a significantly higher species richness in the rhizosphere. In general, there were distinct fewer correlations between the relative abundances of several phyla within the rhizosphere soils which can lead to the hypothesis that the microbial community in the rhizosphere is increasingly dependent on other factors (e.g. plant). In the current study, no correlation could be established between Alphaproteobacteria and any other class in rhizosphere soils whereas in bulk soils several highly significant correlations (positive and negative) could be highlighted. The functional predictions of the community in the soils showed that the predicted cell motility was higher in bulk soil and confirmed that active motility contributes to the movement of microorganisms in soils and enables to move towards nutrient sources^39^, a competence which should be less important in the rhizosphere. Highly significant was also the increase in predicted genes coding for energy-costly ABC-transporters that are responsible for the uptake of small nutrient molecules in the rhizosphere^40^, again pointing to an increased availability of nutrients within the rhizosphere.

Generally, forest soils show on average the highest sink capacity to oxidize CH_4_ of well aerated soils^41^, making it the second largest sink for atmospheric CH_4_ after tropospheric chemical oxidation^42^. Besides abiotic influencing factors for methanotrophs like temperature, pH and soil moisture^43^, atmospheric CH_4_ uptake showed to be sensitive towards vegetation type and plant species^44,45^. Different tree species can affect the methanotrophic community composition and the oxidation capacity of forest soils^16,22^. Thus, the question arose whether the impact of *Larix* and an increased photosynthesis created by additional light is reflected by alterations regarding microorganisms engaged in CH_4_ cycle and the rhizobiome in general. The majority of the archaeal OTUs was identified as representatives belonging to Thaumarchaeota (Nitrososphaeraceae). Due to the lack of isolates of Thermoplasmata not much is known about this lineage, but studies showed that this order occurs not only within humans, where it was initially found^46^ but also in various environmental habitats like soil samples^47^ and animal hosts^48^. Members of Methanomassiliicoccales and Methanocellales dominated the methanogenic community in the studied forest soil. Genomic analysis up to now revealed that Methanomassiliicoccales perform methanogenesis via the methylotrohic pathway^48^ while Methanocellales are hydrogenotrophs^49^. In contrast, methane-oxidizing microorganisms pointed into a major relative abundance of type II methanotrophs, namely representatives of USCα methanotrophs, *Methylosinus* spp, and *Methylocella* spp. Type II methanotrophs are at an advantage in niches where resources are more limiting^50^. Additionally, of USCα methanotrophs which are characterized by *pmoA* genes that distantly cluster from known methanotrophs^51,52^ a draft genome has been recovered from forest soils via metagenomics sequencing methods and shedded light on CH_4_ metabolic potential and environmental adaptions^53^. The derived high affinity for CH_4_ and potential to oxidize atmospheric CH_4_ concentrations was especially proposed for upland soils, mainly for acidic soils^12,15,16^, making USCα a major biological sink of CH_4_ in forest soils^54,55^. Furthermore, methanotrophic Beijerinckiaceae strains to which members of the USCα belong were mostly isolated from peatlands or forest soils^12^. The relative abundance of both, methanogens (not significant) and methanotrophs (significant) was increased in rhizosphere samples although the effects were again very species-specific. Furthermore albeit not significant was the increased relative abundance of methanogens and methanotrophs in the rhizosphere when additional light was exposed.

Besides methanotrophic species, the methylotrophic strain *Methylobacterium* sp. (Methylobacteriaceae—Rhizobiales) which is commonly associated with plants^56^ and known to promote plant growth^57^ was present in the rhizosphere only. Syntrophic relationships between methanotrophs and heterotrophic bacteria have long been proposed but there is still very limited information about how methanotrophs benefit from other bacteria. It was shown that some MOB can be sensitive to methanol and that their activity was initiated after the removal of methanol by *Methylobacterium* sp.^57^. Moreover, *Rhizobium* sp. is believed to provide MOB with essential nutrients and especially with the trace element cobalamin (Vitamin B12)^58,59^. In this study, *Rhizobium* sp. was a dominant member of the rhizospheric community and might have supported the activity and abundance of MOBs. Studies of volatiles in connection with methanotrophs showed that volatile substances also influence the activity and abundance of methanotrophs^60^. Thus, there is increasing evidence that cross-feeding or cross-inhibition between non-methanotrophs and methanotrophs should be considered and opens an interesting field of investigations.

Understanding how tree species affect the composition and distribution of soil microbial communities allows a better comprehension of forest ecosystem functioning. Although the impact of plant species on soil microbial communities seems to be widely documented^4,61,62^, few studies have investigated tree-associated microbial communities by using high-throughput sequencing approaches and there is special lack of knowledge regarding forests, especially covered with *Larix decidua*. Nevertheless, *L. decidua* is a widespread tree species and the second most common coniferous tree species in Austria and a dominant tree species in the Alps^17^. In our study, we focused on the analysis of the rhizosphere and the influence of increasing light on microbial communities and showed that microbial communities within rhizospheres differed significantly from that of bulk soil and that not only taxonomy but also physiological properties changed. The influence of light was detectable in rhizosphere soils, especially in case of prokaryotes and led to the occurrence of several species, e.g. *Pseudomonas* sp. which could point to the increased carbon and nutrient supply due to increased photosynthesis. Trees that grew under increased light treatment were shown to have a higher root biomass (p = 0.048) compared with the plants under normal light conditions, pointing to the possible increased photosynthetic activity. However, the interaction of *Pseudomonas* sp. and methanotrophs was studied by Veraart et al.^60^ who showed that volatiles produced by *Pseudomonas* sp. stimulated the growth of *Methylocystis* sp. but significantly inhibited the methanotrophic activity. Besides, fast growing microbes (e.g. *Massilia*) that are known to utilize plant-derived compounds^63^ significantly increased due to the light treatment as well. Thus, especially r-strategists responded to increased light very rapidly within the treatment period.

Current results offer a detailed insight into the microbiome in the rhizosphere of the ecologically and economically important tree species *Larix decidua* under different light regimes and give insights into the community composition of the root associated methanotrophs and methanogens. Besides the comprehensive analysis of microbial communities in bulk and rhizosphere soil of larch, we could also prove the influence of increasing light intensity on shaping the rhizobiome, which was albeit smaller compared to the effect of the fraction (bulk vs. rhizosphere). The influence of the rhizosphere led to the increase of known plant-associated microorganisms but also to the occurrence of several species not previously known to be associated with the rhizosphere of *L. decidua*.

## Methods

### Site description, soil sampling and determination of basic soil properties

For the present investigation, soil from a forest site in Austria (Tyrol, Seefeld) was chosen. Initially, the forest site was used as a meadow (until 1975), then became a meadow for sheep (until 1987), was abandoned for the last 30 years and is now dominated by larch (*Larix decidua*). Various carbonate and silicate moraines and mixed rocks form the parent material and soil can be classified as eutric endostagnic cambisol according to the world reference base for soil resources (WRB). The forest site lies at an elevation of ~ 1200 m above sea level (a.s.l.), where the annual average air temperature is 5.1 °C and the annual precipitation amount sums up to 1165 mm. For this investigation, soil sampling was conducted in April 2016. For soil sampling, soil material of the soil's upper mineral horizon (at a depth of about 12–20 cm) was taken and sampling was conducted at three replicate sampling sites in the forest field, reflecting site A, B and C. The replicate sites were located with a distance of 5 m from each other. For each replicate site, 5–10 sub-samples were taken and merged which leads to one single sample per replicate site, thus three samples in total. The soil samples were brought to the laboratory in cooled conditions and were sieved to < 4 mm to prepare the mesocosms and < 2 mm for physical and chemical analysis and stored at 4 °C. Dry mass, soil pH, soil organic matter, amount of plant-available ammonium and maximum water holding capacity were determined as described in^64^. Soil electrical conductivity was measured in 1:3.5 (w/v) slurries of soil and deionized water. Total carbon (C_tot_) and total nitrogen (N_tot_) contents of the soils were analyzed on a CN analyzer (Truspec CHN Macro, Leco, MI, USA) using oven-dried (24 h at 105 °C) soil. Dissolved organic carbon (DOC) was quantified in water extracts from 1:5 soil:deionized water (w/v) slurries on a TOC-L analyzer (Shimadzu Corp., Kyoto, Japan).

### Experimental set-up

For the set-up of the mesocosms, planting pots were used to plant the tree seedlings of *Larix decidua* in the sampled forest soil. The 4-year-old seedlings were obtained from the national forest garden in Tyrol/Austria (Landesforstgarten Stams). The growth of the seedlings was performed under unsterile conditions using the same soil for all seeds and was done in the forest garden fields. All seedlings were grown by seeds that came from larches that grew at the same elevation and on the same parent material as the selected study site. After receiving the seedlings, they were carefully cleaned from all the remaining soil from the forest garden by using tweezers and brushes and where then planted into the mesocosm pots. Each planting pot was first filled with a drainage (60 g of polyvinyl chloride pellets) and then received 500 g of soil from the study site. The seedlings were grown in the pots for 20 months before they were used for the experiments. For the plant approaches, eight replicates were created for each replicate site, resulting in 24 pots. For the control approaches (without a tree seedling), six parallels were set up per field site, resulting in another 18 pots. The final set-up of 24 pots with tree seedlings and 18 tree-less pots was halved, with one-half incubated under normal conditions and the other half incubated under increased light exposure. The pots were incubated in a greenhouse, with a day-night light rhythm of 16 h daytime and 8 h night-time. For the investigation of increased light intensity and its effect on microbial communities, greenhouse light intensity (200 µmol m^−2^ s^−1^) was increased for 6 weeks by using a LED light with a 410–460 nm and 610–660 nm light spectrum. By applying additional light, the light intensity in the greenhouse was increased to 1300 µmol m^−2^ s^−1^ for a 16 h photoperiod per day. The temperature in the greenhouse varied between 20 and 25 °C during the day and 10–15 °C during nights. The temperatures chosen correspond to the temperature (extremes) at the investigated forest site during the vegetation period (April–September) and were permanently observed by using thermologgers.

### Sampling of bulk and rhizosphere soil and DNA extraction

At the end of the light experiment, bulk and rhizosphere soil of *L. decidua* were sampled. The sampling procedure followed the procedures described in^7,65^. To obtain bulk soil, soil from the control pots was sampled. Thus after scraping off approx. 5 cm from the soil surface of the pots with a sterile spatula, five soil sub-samples were taken from each pot and merged to one bulk soil sample each pot. In case of planted pots, tree seedlings were removed from the pots and loosely adhering soil was removed by intense hand-shaking. Afterwards, strongly adhering soil was sampled by applying a washing and centrifugation process as described in^7^. For this purpose, the roots were washed in an ultrasonic bath in an Erlenmeyer flask filled with sterile water. The soil–water solution obtained was centrifuged in several steps (each 30 min at 10,000 rpm) to obtain the entire soil sludge^7^. Bulk soil and rhizosphere samples were stored at − 20 °C until DNA extraction. The final sample amount for DNA extraction was 250 mg in case of bulk soil and 400 µl of centrifuged soil slurry from the rhizosphere. For DNA extraction, the NucleoSpin Soil Extraction Kit (Macherey–Nagel, Düren, Germany) was used applying SL1 as the lysis buffer and setting the final elution volume to 50 μl. For the purpose of a quality and quantity control of the DNA extracts, the UV/VIS spectrophotometer NanoDrop 2000c (Thermo Fisher Scientific, Germany) and QuantiFluor dsDNA Dye (Promega, Mannheim, Germany) were used, respectively.

### Amplicon sequencing and sequence processing

Identification of prokaryotic and fungal microorganisms was performed via amplicon sequencing of 16S rRNA and ITS2 genes, respectively. The primers used for sequencing included the primer pair 515f-806r^66^ for amplification of the V4 region of prokaryotes and the primer set gITS7-ITS4^67,68^ targeting the ITS2 region of fungi. PCR amplicons were sequenced using the Illumina MiSeq v2 platform (2 × 250 bp) following Nextera library creation (Microsynth, Balgach, Switzerland). Sequence data processing was performed using the mothur software pipeline v.1.39.0 (64 bit executable) following the Standard Operating Procedures for paired-end sequencing^69^. The raw sequence reads were demultiplexed and adaptor-trimmed. Sequences were further quality-trimmed and denoised in order to remove sequences not fulfilling the required quality score (Phred score of > 25) and containing sequencing errors. For fungal sequences, the ITS2 region was extracted using the ITSx software^70^ to eliminate non-target sequences in advance of any further quality filtering. For prokaryotes and fungi, sequences with ambiguous reads and > 6 homopolymers were removed. The filtered sequenced length was < 240 bp or > 270 bp for Archaea and Bacteria and < 150 bp for fungi. Potential chimeric sequences were removed using VSEARCH^71^. The alignment of the unique prokaryotic sequences was conducted against the SILVA rRNA gene database (release 128)^72^ using kmer searching method. In case of fungi, pairwise comparison with the fungal UNITE ITS database^73^ was performed. Clustering of sequences to operational taxonomic units (OTUs) was performed using the OptiClust algorithm at 97% identity. OTUs with a rarity of < 5 reads were removed. Classification of prokaryotic sequences was performed using the RDP trainset reference database^74^.

### Sequencing data analysis, statistics and calculation of biomarkers

Diversity and richness indices for prokaryotes and fungi were calculated using mothur^69^. Significant differences were ascertained by one-way, multifactorial or multivariate ANOVA. Tukey’s honestly significance test (Tukey’s HSD) and a significance level of 0.05 was used to assess significant differences between variants. In case of non-normality distributed data, non-parametric Mann–Whitney-U Test was applied. For further data analyses, the final OTU table was subsampled to the number of sequences in the smallest sample (30,689 and 40,360 reads for prokaryotes and fungi, respectively). Sub-sampling the OTU tables did not significantly influence the OTU matrix as tested by Mantel test^75^. Significant influence of treatment parameters (fraction, light, site) was tested via analysis of molecular variance (amova)^76^ and ANOSIM (based on Bray–Curtis dissimilarities, permutations = 999)^77^. Non-metric multidimensional scaling (NMDS) based on Bray–Curtis dissimilarity was performed in R (version 3.4.2)^78^ using the package vegan^79^. Biomarker OTUs were identified using the LEfSe command implemented in mothur and by using the public server at *usegalaxy.org*^80^ as previously described in^7^. LEfSe is an algorithm for high-dimensional biomarker discovery and explanation that identifies taxa characterizing the differences between two or more biological conditions (or classes)^81^. OTUs with a linear discriminant analyses (LDA) log score > 3.0 were used for interpretation. OTUs being significantly different abundant between the samples were analyzed using the metastats algorithm as described in^82^. White’s non-parametric *t*-test (two-sided) was used to assess the influence of light and fraction on methanotrophs and p-values were adjusted by the Benjamini–Hochberg method to correct for multiple comparisons (q-values). Statistics and figures were computed and produced in Statistica 12.0 (StatSoft), R (version 3.4.2)^78^, STAMP (version 2.1.3)^83^ and Microsoft Excel.

### Functional predictions: PICRUST and identification of metagenomic biomarkers

For functional predictions, metagenome functional content was predicted from 16S rRNA genes by using the software package PICRUST (phylogenetic investigation of communities by reconstruction of unobserved states) as described in^81^. Therefore, OTUs were picked searching against the Greengenes reference (Greengenes v13.5). Normalization by copy numbers, prediction of the metagenome and categorization by function was performed using the public server at *usegalaxy.org*^80^. Metagenomes were predicted from the copy number normalized 16S rRNA data in PICRUST against the PICRUST-formatted, characterized-protein functional database of KEGG Orthology^84^. Predicted metagenomes were analyzed and visualized with the software STAMP^83^. To identify potential metagenomic biomarkers, the predicted KEGG orthologs that were significantly differently represented in the rhizosphere and bulk soils were detected by using two-sided Welchs’s *t*-test implemented in STAMP^83^ and the LEfSe algorithm^85^ integrated in mothur. For both analysis, the significance threshold was set to 0.05 and the logarithmic LDA score cut-off in LEfSe was set to 2.5. Predicted proteins were classified as KEGG orthologs and summarized on hierarchy level 3.

### Network analysis

Microbial abundance data were converted into a bipartite network and visualized in Cytoscape v3.5.1^86^. For calculating the network, 2000 of the top OTUs were included. To facilitate the visualization, OTUs with fewer than 100 reads were removed from the samples and OTUs with the same tax assignment (on the highest resolvable tax level) were combined. Bipartite network was generated using the samples as source nodes and the OTUs as target nodes, with edges corresponding to positive associations for particular OTUs with specific samples. Thus, the network has samples and OTUs as nodes, and edges were created between the OTUs and the samples in which they were abundant. The edge-weighted spring-embedded layout algorithm implemented in Cytoscape was used to cluster the nodes in which nodes repeal each other and shared edges bring them closer together. Hence, nodes with a large degree of overlap form clusters.

### Phylogenetic analysis

Sequences from selected OTUs classified as ‘Rhizobiales unclassified’ were aligned against 16S rRNA gene sequences from characterized methane-oxidizing bacteria as described in^12^, from a recently reconstructed draft genome of the uncultivated USCα methanotroph^87^ and from methylotrophic species using MAFFT^88^. Phylogenetic placement analyses were calculated only with sequences that had close matches with OTUs. Trees were generated using the geneious plugin for PHYML^89^. PHYML settings were: GTR with Chi^2^ statistics, 4 substitution rate categories and the settings to optimize Topology/length/rate and the BEST topology search option selecting the best topology of NNI and SPR search.

## Supplementary Information

Supplementary Information.

## Data Availability

All sequence data obtained in this study have been made available in the National Center for Biotechnology Information (NCBI) Sequence Read Archive (SRA) and are accessible through the SRA accession number SRP155730.
