# Supplementary material for: Microbial community composition in the rhizosphere of Larix decidua under different light regimes with additional focus on methane cycling microorganisms
Source: Sci Rep. 2020 Dec 18;10:22324. doi: 10.1038/s41598-020-79143-y (PMC7749151; doi:10.1038/s41598-020-79143-y)
Supplement: Supplementary file 1 — Supplementary Information. [file 41598_2020_79143_MOESM1_ESM.pdf]

# Microbial community composition in the rhizosphere of *Larix decidua* under different light regimes with additional focus on methane cycling microorganisms

**Nadine Praeg[a]* and Paul Illmer[a]**

[a]Universität Innsbruck, Department of Microbiology, Technikerstrasse 25d, A-6020 Innsbruck, Austria

*Corresponding author: Nadine Praeg

## Supplementary Information

### Supplementary Information Fig. S1-S7

### Supplementary Information Table S1+S2

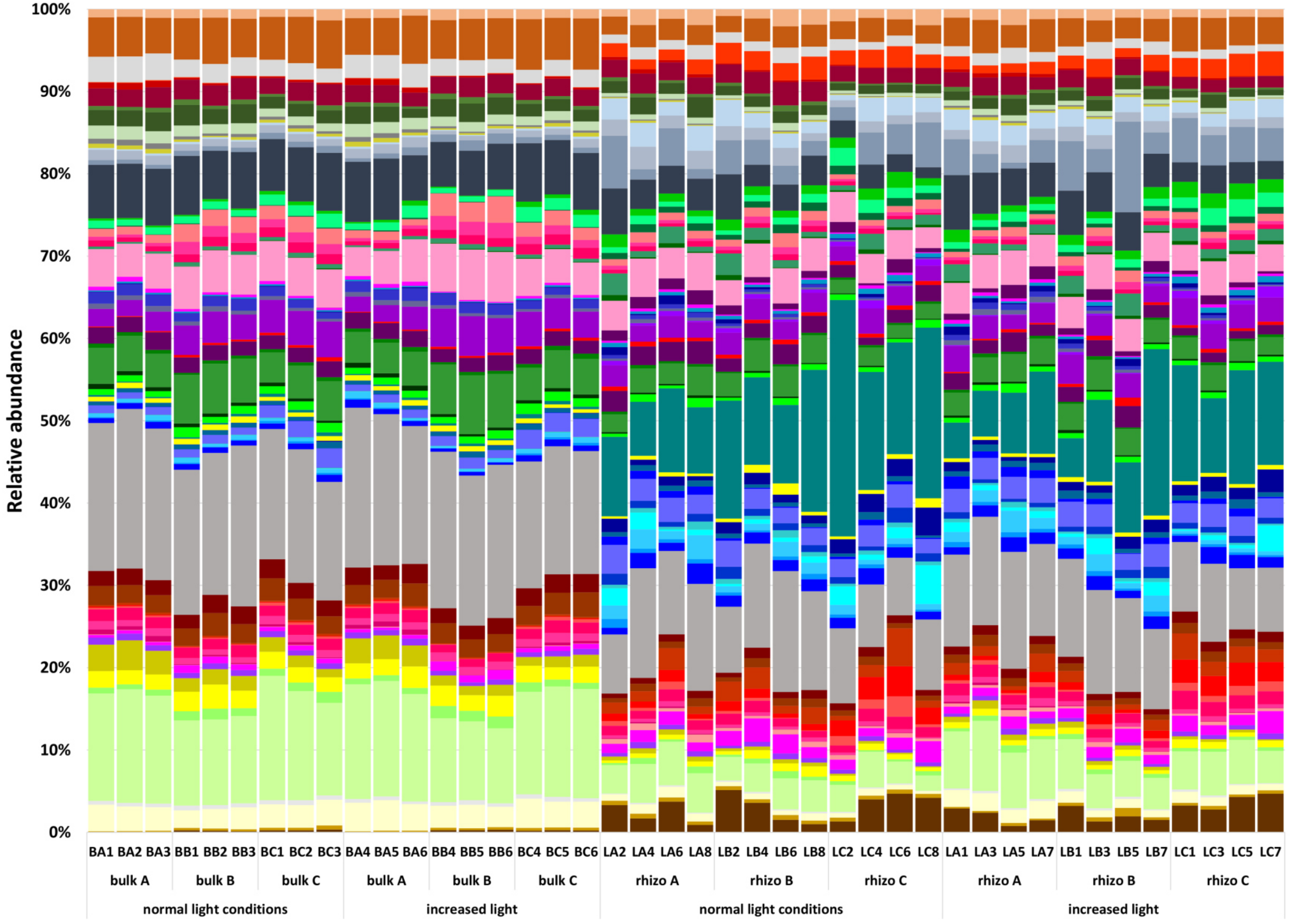


**Figure S1:** Community composition of prokaryotes on genus level representing most abundant genera (98% of relative abundance) in bulk and rhizosphere (rhizo) soils at normal and increased light conditions. A,B,C represent the replicate forest sites. BA, BB, BC stand for bulk soils on site replicate A, B and C. LA, LB, LC stand for rhizosphere soil of *L. decidua* (L) on site replicate A, B and C.

| Phylum | Class | Order | Family | Genus |
|---|---|---|---|---|
| **Verrucomicrobia** | Subdivision3 | Subdivision3 unclassified | Subdivision3 unclassified | Subdivision3 unclassified |
| | Spartobacteria | Spartobacteria unclassified | Spartobacteria unclassified | Spartobacteria unclassified |
| | Proteobacteria unclassified | Proteobacteria unclassified | Proteobacteria unclassified | Proteobacteria unclassified |
| **Proteobacteria** | Gammaproteobacteria | Xanthomonadales | Sinobacteraceae | *Steroidobacter spp.* |
| | | | | Sinobacteraceae unclassified |
| | | Gammaproteobacteria unclassified | Gammaproteobacteria unclassified | Gammaproteobacteria unclassified |
| | Deltaproteobacteria | Myxococcales | Polyangiaceae | Polyangiaceae unclassified |
| | | | Myxococcales unclassified | Myxococcales unclassified |
| | | Deltaproteobacteria unclassified | Deltaproteobacteria unclassified | Deltaproteobacteria unclassified |
| | Betaproteobacteria | Rhodocyclales | Rhodocyclaceae | Rhodocyclaceae unclassified |
| | | Nitrosomonadales | Nitrosomonadaceae | *Nitrosospira* spp. |
| | | Burkholderiales | Comamonadaceae | Comamonadaceae unclassified |
| | | | Burkholderiaceae | Burkholderiaceae unclassified |
| | | | | *Burkholderia* spp. |
| | | Betaproteobacteria unclassified | Betaproteobacteria unclassified | Betaproteobacteria unclassified |
| | Alphaproteobacteria | Sphingomonadales | Sphingomonadaceae | *Sphingomonas* spp. |
| | | | | Sphingomonadaceae unclassified |
| | | | | *Rhizorhabdus* spp. |
| | | Rhodospirillales | Rhodospirillales unclassified | Rhodospirillales unclassified |
| | | | Rhodospirillaceae | Rhodospirillaceae unclassified |
| | | | Reyranella | Reyranella unclassified |
| | | | Acetobacteraceae | Acetobacteraceae unclassified |
| | | Rhizobiales | Xanthobacteraceae | *Labrys* spp. |
| | | | Rhizobiales unclassified | Rhizobiales unclassified |
| | | | Rhizobiaceae | *Rhizobium* spp. |
| | | | Phyllobacteriaceae | Phyllobacteriaceae unclassified |
| | | | | *Mesorhizobium* spp. |
| | | | Hyphomicrobiaceae | *Rhodomicrobium* spp. |
| | | | | *Pedomicrobium* spp. |
| | | | | *Hyphomicrobium* spp. |
| | | | | *Devosia* spp. |
| | | | Bradyrhizobiaceae | *Bradyrhizobium* spp. |
| | | Caulobacterales | Caulobacteraceae | *Phenylobacterium* spp. |
| | | Alphaproteobacteria unclassified | Alphaproteobacteria unclassified | Alphaproteobacteria unclassified |
| **Planctomycetes** | Planctomycetia | Planctomycetales | Planctomycetaceae | *Singulisphaera* spp. |
| | | | | Planctomycetaceae unclassified |
| | | | | *Pirellula* spp. |
| **Gemmatimonadetes** | Gemmatimonadetes | Gemmatimonadales | Gemmatimonadaceae | *Gemmatimonas* spp. |
| **Candidatus Saccharibacteria** | Candidatus Saccharibacteria | Candidatus Saccharibacteria unclassified | Candidatus Saccharibacteria unclassified | Candidatus Saccharibacteria unclassified |
| **Candidate division WPS-1** | Candidate division WPS-1 | Candidate division WPS-1 unclassified | Candidate division WPS-1 unclassified | Candidate division WPS-1 unclassified |
| **Bacteroidetes** | Sphingobacteriia | Sphingobacteriales | Sphingobacteriaceae | *Mucilaginibacter* spp. |
| | | | Chitinophagaceae | *Terrimonas* spp. |
| | | | | *Ferruginibacter* spp. |
| | | | | Chitinophagaceae unclassified |
| | | | | *Chitinophaga* spp. |
| | Flavobacteriia | Flavobacteriales | Cryomorphaceae | *Fluviicola* spp. |
| | | Cytophagales | Ohtaekwangia | Ohtaekwangia unclassified |
| | | | Cytophagaceae | Cytophagaceae unclassified |
| | Cytophagia | | Chryseolinea | Chryseolinea unclassified |
| | Bacteroidetes unclassified | Bacteroidetes unclassified | Bacteroidetes unclassified | Bacteroidetes unclassified |
| **Bacteria unclassified** | Bacteria unclassified | Bacteria unclassified | Bacteria unclassified | Bacteria unclassified |
| **Actinobacteria** | Actinobacteria | Actinobacteria unclassified | Actinobacteria unclassified | Actinobacteria unclassified |
| | | Gaiellales | Gaiellaceae | *Gaiella* spp. |
| | | Actinomycetales | Streptomycetaceae | Streptomycetaceae unclassified |
| | | | | *Streptomyces* spp. |
| | | | Nocardioidaceae | *Kribbella* spp. |
| | | | Mycobacteriaceae | *Mycobacterium* spp. |
| | | | Micromonosporaceae | Micromonosporaceae unclassified |
| | | | Microbacteriaceae | *Agromyces* spp. |
| | | | Actinospicaceae | *Actinospica* spp. |
| | | | Actinomycetales unclassified | Actinomycetales unclassified |
| | | Acidimicrobiales | Acidimicrobiales unclassified | Acidimicrobiales unclassified |
| **Acidobacteria** | Acidobacteria Gp17 | Gp17 | Gp17 unclassified | Gp17 unclassified |
| | Acidobacteria Gp16 | Gp16 | Gp16 unclassified | Gp16 unclassified |
| | Acidobacteria Gp7 | Gp7 | Gp7 unclassified | Gp7 unclassified |
| | Acidobacteria Gp6 | Gp6 | Gp6 unclassified | Gp6 unclassified |
| | Acidobacteria Gp5 | Gp5 | Gp5 unclassified | Gp5 unclassified |
| | Acidobacteria Gp4 | Gp4 | Gp4 unclassified | Gp4 unclassified |
| | | Granulicella | Granulicella unclassified | Granulicella unclassified |
| | Acidobacteria Gp1 | Acidobacteria Gp1 unclassified | Acidobacteria Gp1 unclassified | Acidobacteria Gp1 unclassified |

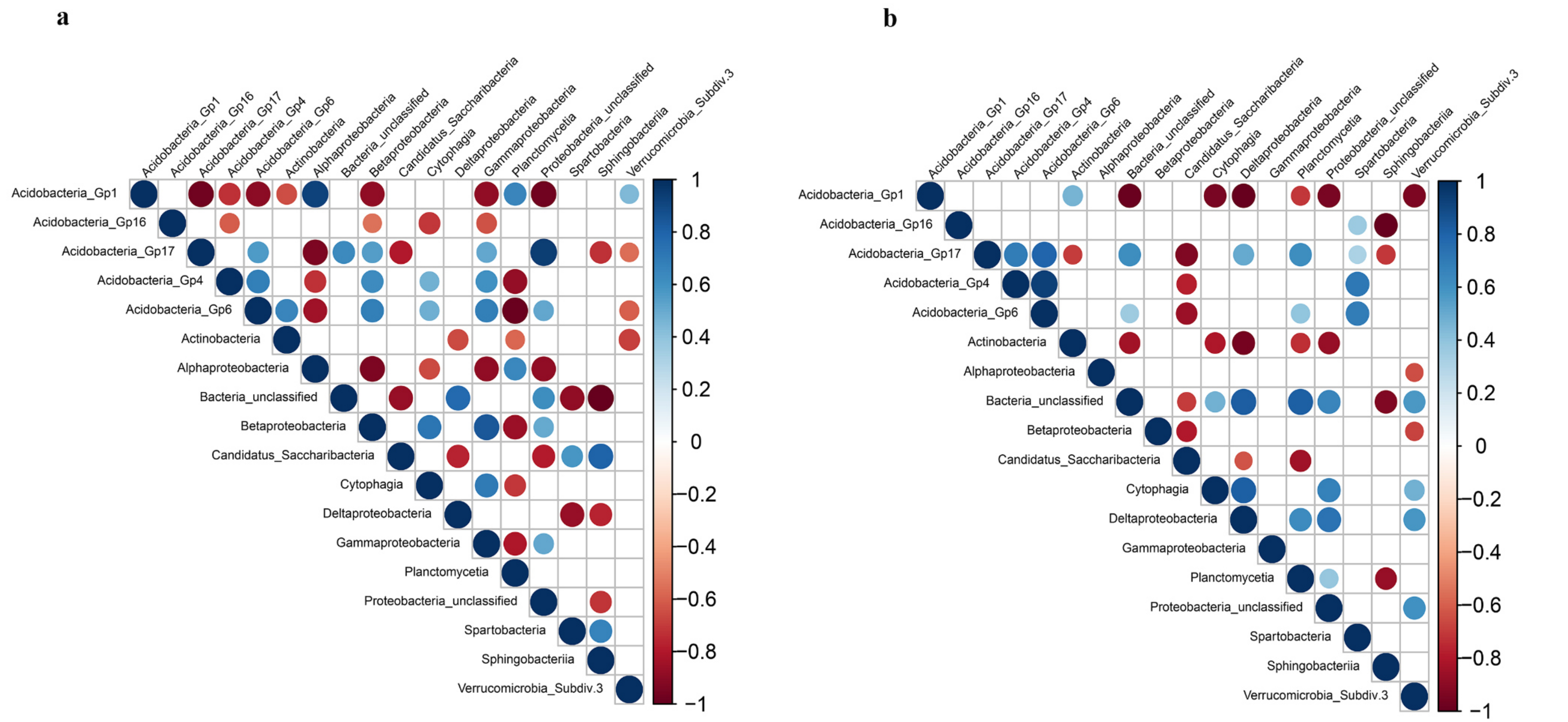


**Figure S2:** Spearman's rank correlation matrix of bacterial classes in bulk (a) and rhizosphere (b) soils. Strong correlations are indicated by large circles and weak correlations are indicated by small circles. The colours of the scale bar denote the nature of the correlation with 1 indicating perfect positive correlation (dark blue) and -1 indicating perfect negative correlation (dark red) between two bacterial classes. Only significant correlations are shown ($p < 0.05$).

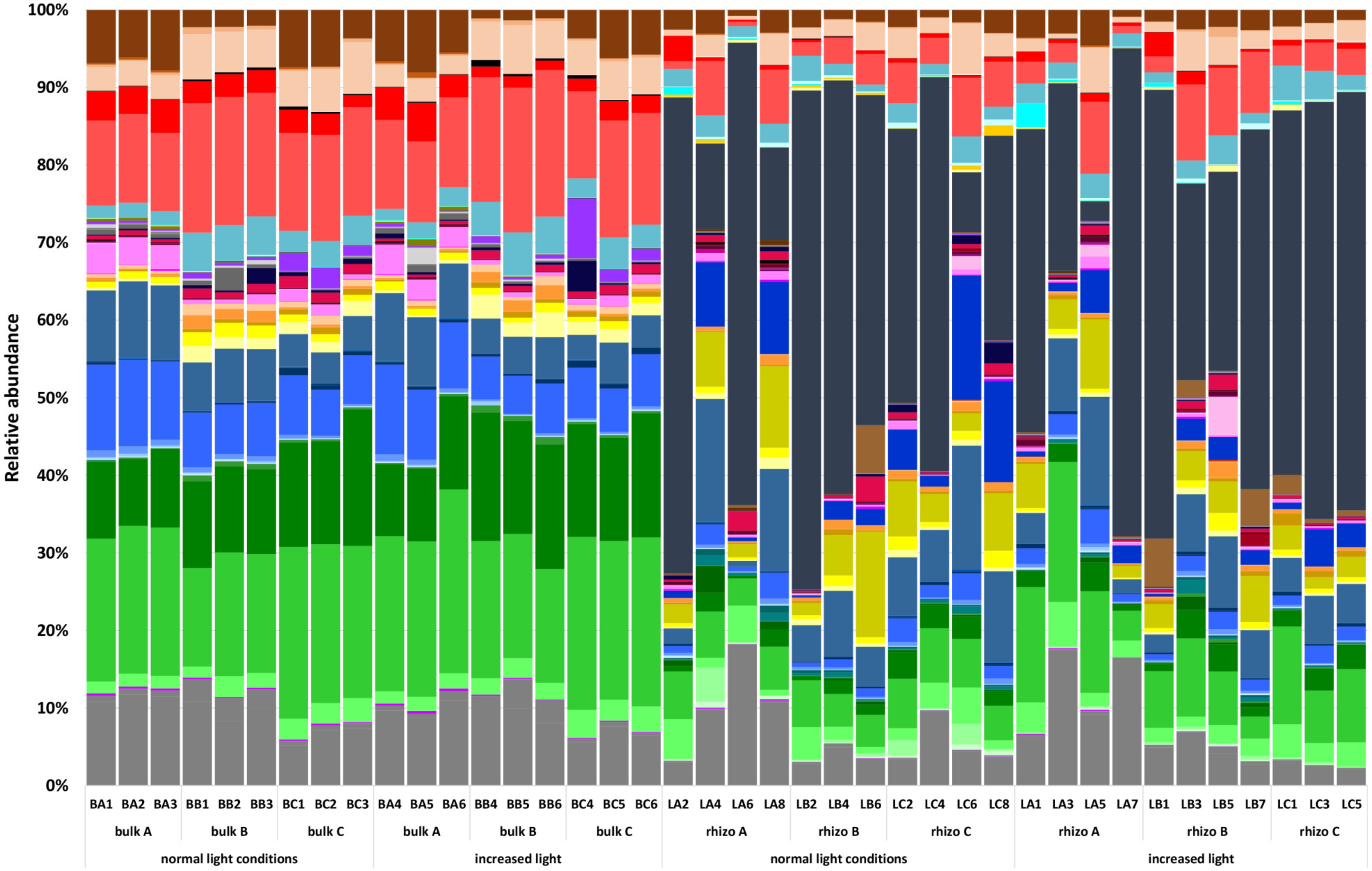


**Figure S3:** Community composition of fungi on genus level representing most abundant genera (99% of relative abundance) in bulk and rhizosphere (rhizo) soils at normal and increased light conditions. A,B,C represent the replicate forest sites. BA, BB, BC stand for bulk soils on site replicate A, B and C. LA, LB, LC stand for rhizosphere soil of *L. decidua* (L) on site replicate A, B and C. LB8 and LC7 were excluded for the description of the fungal community composition as the samples did not meet the quality requirements.

| Phylum | Class | Order | Family | Genus |
|---|---|---|---|---|
| **Zygomycota** | Zygomycota class Incertae sedis | Mucorales | Umbelopsidaceae | *Umbelopsis* spp. |
| | | | Mucoraceae | *Mucor* spp. |
| | | | | Mucoraceae unclassified |
| | | Mortierellales | Mortierellaceae | *Mortierella* spp. |
| | | Kickxellales | Kickxellaceae | *Ramicandelaber* spp. |
| **Basidiomycota** | Basidiomycota unclassified | Basidiomycota unclassified | Basidiomycota unclassified | Basidiomycota unclassified |
| | Wallemiomycetes | Geminibasidiales | Geminibasidiaceae | *Geminibasidium* spp. |
| | Tremellomycetes | Filobasidiales | Filobasidiaceae | *Cryptococcus* spp. |
| | Microbotryomycetes | Sporidiobolales | unclassified Sporidiobolales | unclassified Sporidiobolales |
| | | | Sporidiobolales family Incertae sedis | *Rhodotorula* spp. |
| | Agaricostilbomycetes | Agaricostilbales | Chionosphaeraceae | *Kurtzmanomyces* spp. |
| | | Trechisporales | unclassified Trechisporales | unclassified Trechisporales |
| | | | Hydnodontaceae | *Trechispora* spp. |
| | | Thelephorales | Thelephoraceae | *Tomentella* spp. |
| | | Sebacinales | Sebacinaceae | Sebacinaceae unclassified |
| | | Boletales | Suillaceae | *Suillus* spp. |
| | | | Gomphidiaceae | unclassified Gomphidiaceae |
| | | Auriculariales | unclassified Auriculariales | unclassified Auriculariales |
| | | Agaricales | Agaricales unclassified | Agaricales unclassified |
| | | | Tricholomataceae | unclassified Tricholomataceae |
| | | | Mycenaceae | Mycenaceae unclassified |
| | | | Inocybaceae | *Inocybe* spp. |
| | | | Hygrophoraceae | *Hygrocybe* spp. |
| | | | Agaricaceae | *Echinoderma* spp. |
| | Agaricomycetes | Agaricomycetes unclassified | Agaricomycetes unclassified | Agaricomycetes unclassified |
| **Ascomycota** | Ascomycota unclassified | Ascomycota unclassified | Ascomycota unclassified | Ascomycota unclassified |
| | | Xylariales | Hyponectriaceae | unclassified Hyponectriaceae |
| | | | Amphisphaeriaceae | unclassified Amphisphaeriaceae |
| | Sordariomycetes | Sordariales | Sordariales unclassified | Sordariales unclassified |
| | | Hypocreales | Hypocreales unclassified | Hypocreales unclassified |
| | | | | *Stachybotrys* spp. |
| | | | | *Ilyonectria* spp. |
| | | | Hypocreales family Incertae sedis | *Acremonium* spp. |
| | | | | *Hypocrea* spp. |
| | | | Hypocreaceae | Hypocreaceae unclassified |
| | | | Cordycipitaceae | Cordycipitaceae unclassified |
| | | | Clavicipitaceae | *Metarhizium* spp. |
| | Pezizomycetes | Pezizales | Pyronemataceae | *Wilcoxina* spp. |
| | Leotiomycetes | Leotiomycetes order Incertae sedis | Leotiomycetes family Incertae sedis | *Meliniomyces* spp. |
| | | | | *Leohumicola* spp. |
| | | Helotiales | Helotiales unclassified | Helotiales unclassified |
| | | | | *Leptodontidium* spp. |
| | | | Helotiales family Incertae sedis | *Cadophora* spp. |
| | | | Dermateaceae | Dermateaceae unclassified |
| | | Leotiomycetes unclassified | Leotiomycetes unclassified | Leotiomycetes unclassified |
| | Eurotiomycetes | Eurotiales | Trichocomaceae | Trichocomaceae unclassified |
| | | | | *Penicillium* spp. |
| | | | | *Paecilomyces* spp. |
| | | Chaetothyriales | Chaetothyriales unclassified | Chaetothyriales unclassified |
| | | | Herpotrichiellaceae | *Exophiala* spp. |
| | | | | *Cladophialophora* spp. |
| | | | | Herpotrichiellaceae unclassified |
| | | Eurotiomycetes unclassified | Eurotiomycetes unclassified | Eurotiomycetes unclassified |
| | | Pleosporales | Pleosporales family Incertae sedis | *Phoma* spp. |
| | | | | Pleosporales family Incertae sedis unclassified |
| | | | Pleomassariaceae | Pleomassariaceae unclassified |
| | | Hysteriales | Gloniaceae | *Cenococcum* spp. |
| | | Dothideomycetes order Incertae sedis | Pseudeurotiaceae | unclassified Pseudeurotiaceae |
| | | | Myxotrichaceae | *Oidiodendron* spp. |
| | | | | Myxotrichaceae unclassified |
| | | Dothideales | Dothioraceae | *Aureobasidium* spp. |
| | Dothideomycetes | Capnodiales | Davidiellaceae | Davidiellaceae unclassified |
| | Ascomycota class Incertae sedis | Ascomycota order Incertae sedis | Ascomycota family Incertae sedis | *Tetrachaetum* spp. |
| | | | | *Chalara* spp. |
| **Fungi unclassified** | Fungi unclassified | Fungi unclassified | Fungi unclassified | Fungi unclassified |

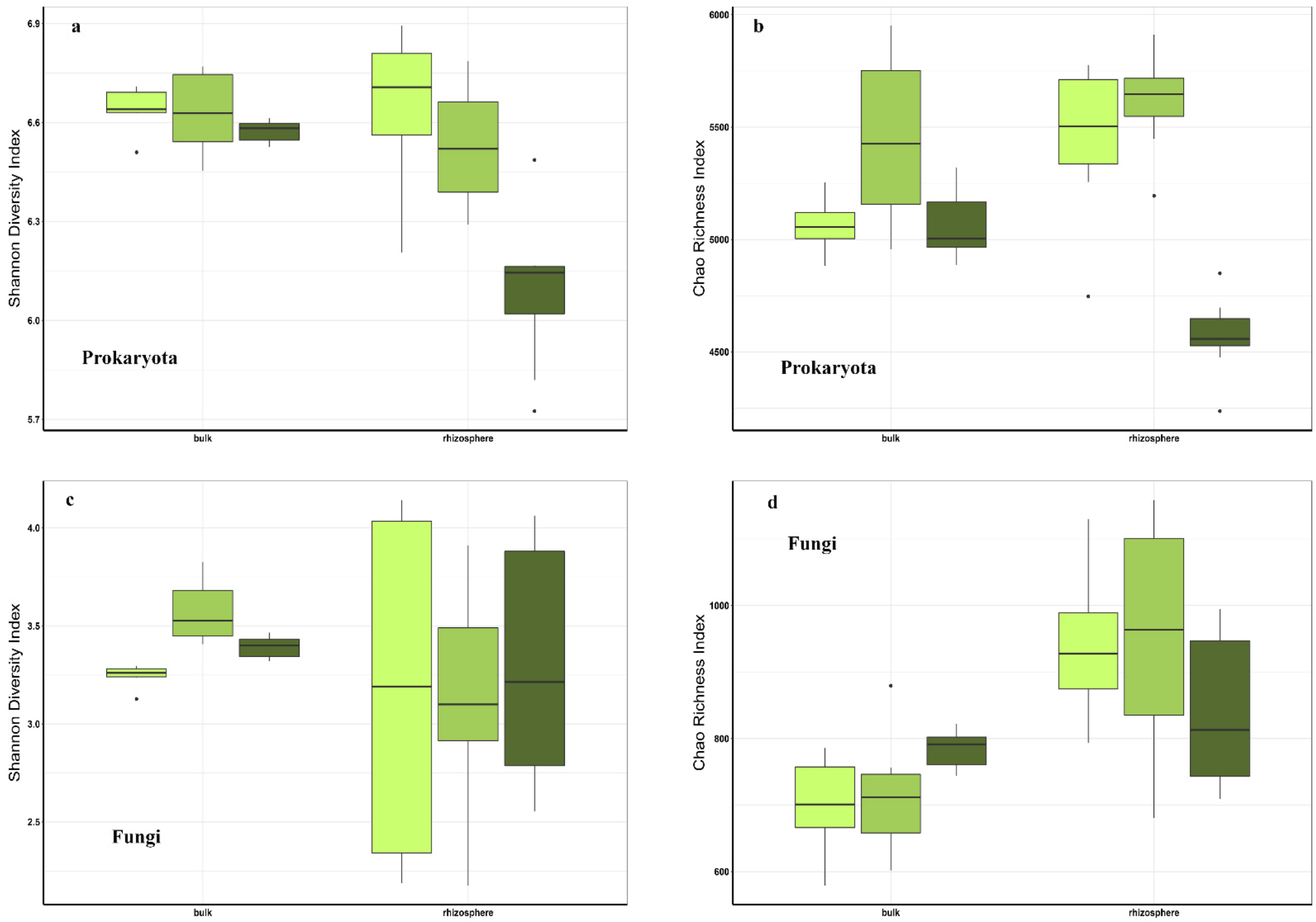


**Figure S4:** Shannon–Wiener Diversity Index (H') (**a**) and Chao Richness Index (**b**) of prokaryotic communities and Shannon-Wiener Diversity Index (H') (**c**) and Chao Richness Index (**d**) of fungal communities in all bulk and rhizosphere soils of *Larix decidua* (L). The different green colors refer to the replicate sites (A, B, C) in the forest. Boxes represent 25–75% of values, black lines medians (n = 3), and whiskers 1.5 interquartile ranges.

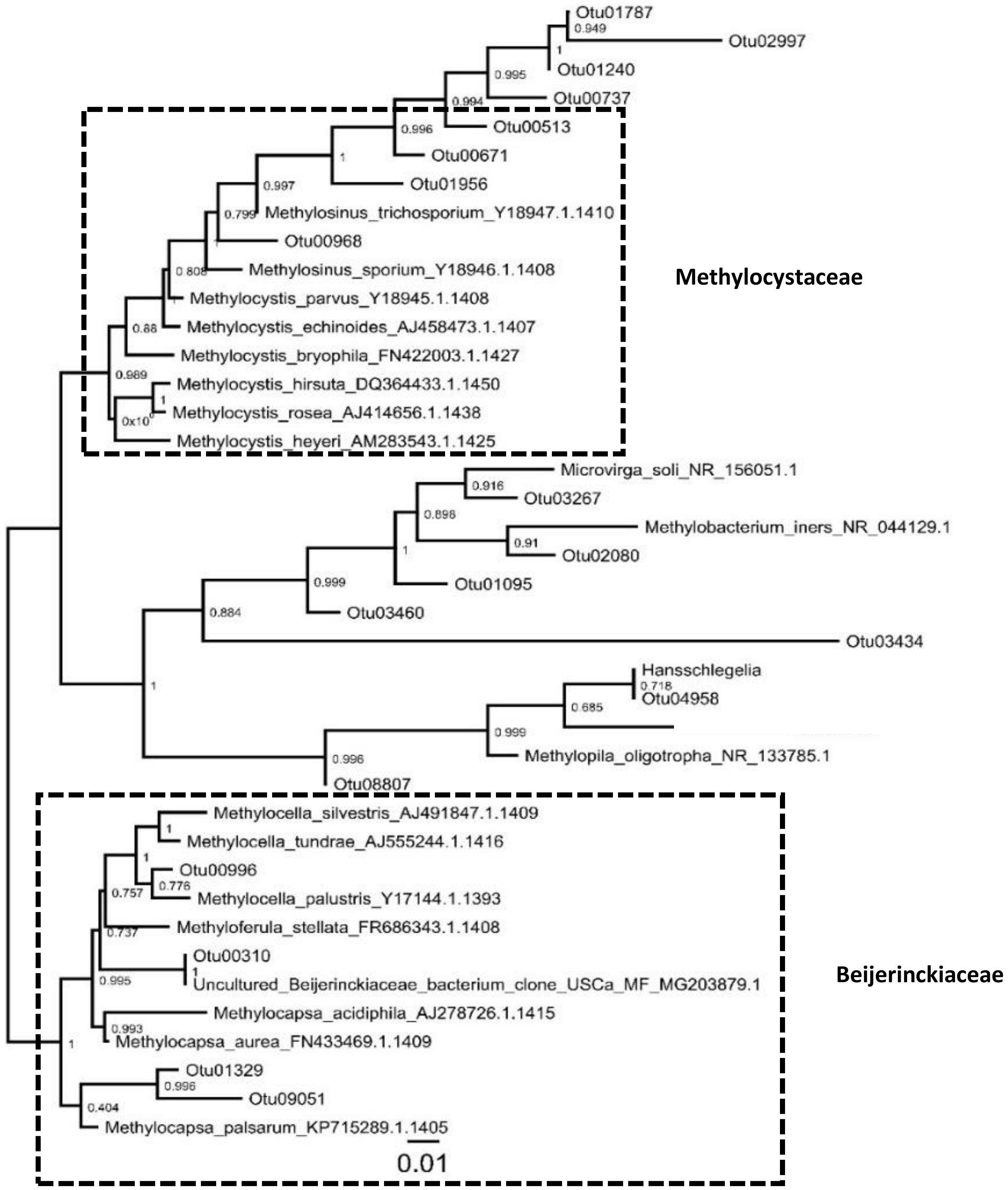


**Figure S5:** Unrooted Maximum likelihood tree (PhyML) showing the relationship between selected OTUs that were classified as 'Rhizobiales unclassified' as the highest taxonomic resolution and selected reference methanotrophs (and methylotrophic species) within Alphaproteobacteria known from the literature and a reconstructed draft genome of the uncultivated upland soil cluster α (USCα) methanotroph (Pratscher et al., 2018). The scale bar represents 1% nucleotide substitution per site and numbers at nodes of the tree indicate Chi2 values.

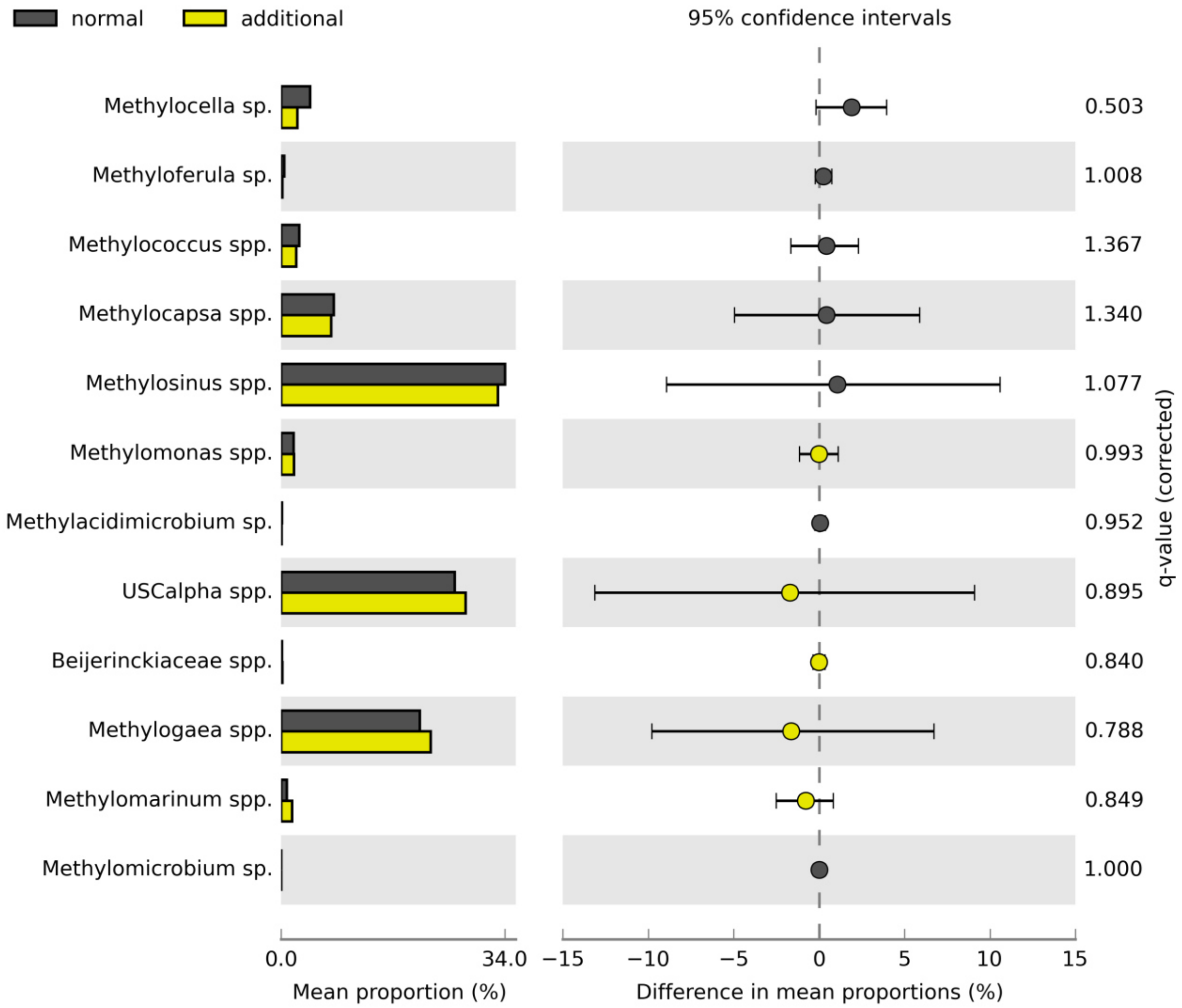


**Figure S6**: Extended error bar plot showing mean proportions [%] of methanotrophic genera in rhizosphere soils of *L. decidua* and the respective differences in mean proportions [%] according to normal (grey) and additional (yellow) light conditions. q-values are adjusted p-values following Benjamini-Hochberg method for multiple test correction.

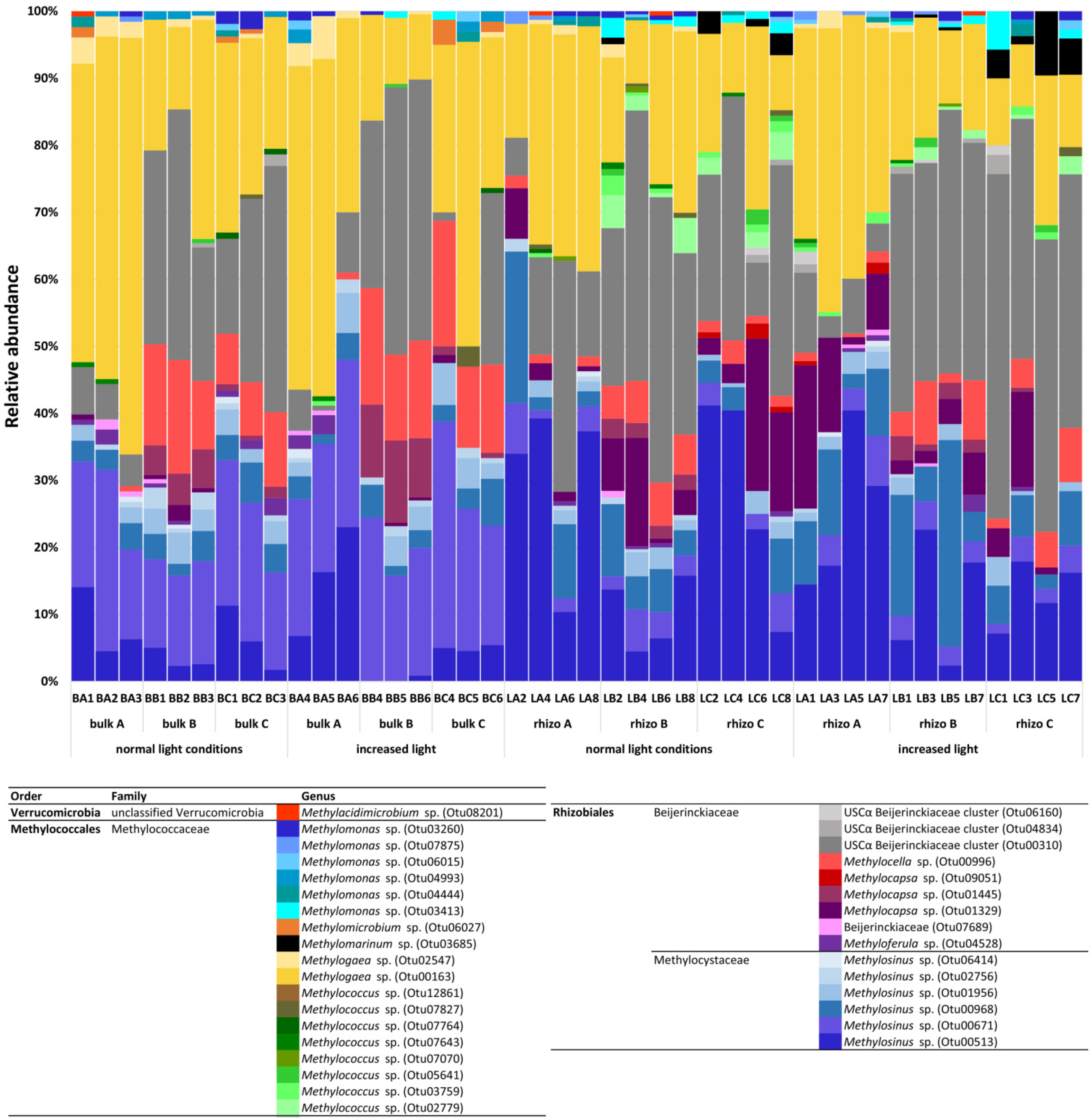


**Figure S7:** Community composition of methanotrophs on OTU level in bulk and rhizosphere (rhizo) soils at normal and increased light conditions. A,B,C represent the replicate forest sites. BA, BB, BC stand for bulk soils on site replicate A, B and C. LA, LB, LC stand for rhizosphere soil of *L. decidua* (L) on site replicate A, B and C. OTU, operational taxonomic unit.

**Table S1:** Prokaryotic and fungal community description with dominant phyla, classes, orders and families including the respective relative abundances [%] both, in bulk and rhizosphere soils. Significant differences regarding dominant phyla, classes, families and species are indicated in blue (bulk) and red (rhizosphere). Black colors point to no significant differences between bulk and rhizosphere soils. 1) Abundances are given in the order: bulk, rhizosphere, 2) Significant differences regarding dominant phyla, classes, families and species are indicated in blue (bulk) and red (rhizosphere). Black colors point to no significant differences between bulk and rhizosphere soils.

| Domain or kingdom | dominant phyla | relative abundance [%][1, 2)] | dominant class | class abundance [%][1, 2)] | dominant order | order abundance [%][1, 2)] | dominant family[2)] | dominant or exclusive species[2)] |
|---|---|---|---|---|---|---|---|---|
| Bacteria | **Proteobacteria** | **34, 37.3** | α-Proteobacteria | 17, 18 | Rhizobiales | 9.9, 10.1 | Bradyrhizobiaceae | *Bradyrhizobium* sp. |
| | | | | | | | Hyphomicrobiaceae | *Pedomicrobium* sp. |
| | | | | | | | Rhizobiaceae | *Rhizobium* sp. |
| | | | | | Rhodospirillales | 3.7, 3.8 | Acetobacteriaceae | *Acidocella* sp. |
| | | | | | | | Rhodospirillaceae | *Inquilinus* sp. |
| | | | | | Sphingomonadales | 1.3, 2.3 | Erythrobacteraceae, Sphingomonadaceae | *Altererythrobacter* sp. |
| | | | β-Proteobacteria | 8, 10 | Burkholderiales | 1.9, 6.5 | Burkholderiaceae | *Burkholderia* sp. |
| | | | | | | | Comamonadaceae | |
| | | | | | | | Oxalobacteraceae | |
| | | | | | Betaproteobacteria unclassified | 5.6, 3.1 | | |
| | | | γ-Proteobacteria | 3.6, 5,4 | Xanthomonadales | 1.4, 3.4 | Xanthomonadaceae | *Rhodanobacter* sp., *Pseudoxanthomonas* sp. |
| | | | | | | | Sinobacteraceae | *Steroidobacter* sp. |
| | | | δ-Proteobacteria | 3.5, 2.4 | Myxococcales | 2.3, 1.5 | | |
| | **Acidobacteria** | **22.2, 11.6** | Acidobacteria_Gp6 | 10.6, 3.9 | | | | |
| | | | Acidobacteria_Gp4 | 2.6, 0.9 | | | | |
| | | | Acidobacteria_Gp1 | 0.1, 2.3 | | | Acidobacteria_Gp1 | *Granulicella* sp. |
| | | | Acidobacteria_Gp16 | 1.8, 0.6 | | | | |
| | | | Acidobacteria_Gp17 | 1.7, 0.4 | | | | |
| | **Bacteria unclassified** | **15.1, 8.8** | Bacteria unclassified | 15, 9 | | | | |
| | **Actinobacteria** | **9.8, 11.0** | Actinobacteria | 9, 10.6 | Actinomycetales | 4.2, 8.2 | Streptomycetaceae | *Streptomyces* sp. |
| | | | | | | | Mycobacteriaceae | *Mycobacterium* sp. |
| | | | | | | | Micromonosporaceae | *Actinoplanes* sp. |
| | | | | | | | Nocardioidaceae | *Nocardioides* sp., *Kribbella* sp. |
| | | | | | Gaiellales | 2.1, 0.8 | Gaiellaceae | *Gaiella* sp. |
| | | | | | Acidimicrobiales | 1.1, 0.7 | Acidimicrobiaceae | *Ilumatobacter* sp. |
| | **Bacteroidetes** | **3.6, 10.17** | Sphingobacteriia | 2, 6 | Sphingobacteriales | 2, 6 | Chitinophagaceae | *Chitinophaga* sp. |
| | | | | | | | Sphingobacteriaceae | *Mucilaginibacter* sp. |
| | | | Cytophagia | 0.7, 2.6 | Cytophagales | 0.7, 2.6 | Cytophagaceae | *Dyadobacter* sp. |
| | Cd. Saccharibacteria | **0.3, 10** | *Cd.* Saccharibacteria | 0.3, 10 | | | | |
| | **Verrucomicrobia** | **5.8, 4.6** | Spartobacteria | 4.5, 2.4 | Spartobacteria unclassified | 4.5, 2.4 | | |
| | | | Opitutae | 0.1, 0.5 | Opitutales | 0.1, 0.5 | Opitutaceae | *Opitutus* sp. |
| | | | Subdivsion 3 | 0.9, 1.1 | Subdivision3 | 0.9, 1.1 | | |

| | | | | | | | | |
|---|---|---|---|---|---|---|---|---|
| | | | | | unclassified | | | |
| | **Planctomycetes** | **5.7, 3.2** | Planctomycetia | 5.7, 3.2 | Planctomycetales | 5.7, 3.2 | Planctomycetaceae | *Singulisphaera* sp., *Pirellula* sp. |
| | **Firmicutes** | **0.8, 0.7** | Bacilli | 0.8, 0.7 | Bacillales | 0.8, 0.7 | | |
| | **Gemmatimonadetes** | **0.8, 0.6** | Gemmatimonadetes | 0.8, 0.6 | Gemmatimonadales | 0.8, 0.6 | | |
| | **Chloroflexi** | **0.5, 0.8** | Ktedonobacteria | 0.1, 0.5 | Ktedonobacterales | 0.1, 0.5 | | |
| | **candidate_division WPS-1** | **0.5, 0.5** | candidate_division WPS-1 unclassified | 0.5, 0.5 | | | | |
| | **Armatimonadetes** | **0.2, 0.4** | Armatimonadia | 0.2, 0.4 | | | | |
| **Archaea** | **Thaumarchaeota** | **0.03, 0.06** | Nitrososphaerales | 0.03, 0.06 | Nitrososphaeria | 0.01, 0.03 | Nitrososphaeraceae | |
| | **Euryarchaeota** | **0.02, 0.02** | | | | | | |
| | | | | | | | | |
| **Fungi** | **Ascomycota** | **56.5, 38.9** | Dothideomycetes | 33.5, 15.0 | Dothideomycetes order incertae sedis | 33, 12 | Myxotrichaceae | *Oidiodendron* sp. |
| | | | | | | | Pseudeurotiaceae | |
| | | | | | Pleosporales | 0.5, 1.35 | Pleosporales family incertae sedis | *Phoma* sp., *Setomelanomma* sp. |
| | | | | | | | Pleomassariaceae | |
| | | | | | Dothideales | 0, 0.7 | Dothioraceae | *Aureobasidium* sp. |
| | | | | | Capnodiales | 0.05, 0.3 | Davidiellaceae | |
| | | | | | Hysteriales | 0.01, 0.5 | Gloniaceae | *Cenococcum* sp. |
| | | | Eurotiomycetes | 15, 10.6 | Eurotiales | 6.6, 7.5 | Trichocomaceae | *Penicillium* sp. |
| | | | | | Chaetothyriales | 8.3, 2.5 | Herpotrichiellaceae | *Exophiala* sp. |
| | | | Leotiomycetes | 4.6, 7.3 | Helotiales | 2.5, 6.7 | Helotiales family incertae sedis | *Cadophora* sp. |
| | | | Sordariomycetes | 2.3, 2.0 | Hypocreales | 2.0, 1.7 | Hypocreaceae | |
| | | | Pezizomycetes | 0.01, 3.7 | Pezizales | 0.01, 3.7 | Pyronemataceae | *Wilcoxina* sp. |
| | **Basidiomycota** | **23.9, 49.4** | Agaricomycetes | 3.7, 40.6 | Boletales | 0.07, 38.6 | Suillaceae | *Suillus* sp. |
| | | | | | Agaricales | 2.5, 0.4 | Hygrophoraceae | *Hygrocybe* sp. |
| | | | | | Trechisporales | | | |
| | | | Tremellomycetes | 13.6, 4.7 | Filobasidiales | 8.8, 2.6 | Filobasidiaceae | |
| | | | Tremellomycetes Microbotryomycetes | | Tremellales | 4.8, 2.1 | Tremellales family incertae sedis | *Cryptococcus* sp. |
| | | | | 3.5, 2.8 | Sporidiobolales | 3.3, 2.7 | | |
| | | | Wallemiomycetes | 2.7, 0.8 | Geminibasidiales | 2.7, 0.8 | Geminibasidiaceae | *Geminibasidium* sp. |
| | **Zygomycota** | **9. 5, 5** | Zygomycota class incertae sedis | 9.5, 5.0 | Mortierellales | 4.4, 2.6 | Mortierellaceae | *Mortierella* sp. |
| | | | | | Mucorales | 5.1, 2.3 | Umbelopsidaceae | *Umbelopsis* sp. |

**Table S2:** Site description and physical and chemical soil characteristics of the replicate study sites (soil site) including dry matter content (DM), pH, electrical conductivity (EC), soil organic matter content (OM), dissolved organic carbon (DOC), maximum water holding capacity (MWHC), total carbon (C) and nitrogen (N) content, carbon/nitrogen ratio (C/N), and plant-available ammonium concentration ($NH_4^+$-N). Data represent means ± standard deviations (*n* = 3) in italics.

| ***Soil site*** | **Replicate site** | **DM [g g$^{-1}$]** | **pH** | **EC [µS m$^{-1}$]** | **OM [g g$^{-1}$ DM]** | **DOC [µg g$^{-1}$ DM]** | **MWHC [g g$^{-1}$ DM]** | **C [%]** | **N [%]** | **C/N** | **$NH_4^+$-N [µg g$^{-1}$ DM]** |
|---|---|---|---|---|---|---|---|---|---|---|---|
| ***Gschwandtkopf Seefeld/Austria*** | **A** | 0.67 | 6.86 | 82.67 | 0.11 | 135.40 | 0.98 | 5.45 | 0.28 | 19.57 | 5.29 |
| | | *0.00* | *0.05* | *3.51* | *0.00* | *19.60* | *0.04* | *0.36* | *0.01* | *1.52* | *0.70* |
| | **B** | 0.65 | 6.34 | 103.67 | 0.11 | 165.94 | 1.14 | 5.53 | 0.26 | 21.79 | 11.82 |
| | | *0.00* | *0.05* | *0.58* | *0.00* | *2.13* | *0.01* | *0.42* | *0.01* | *2.21* | *1.20* |
| | **C** | 0.71 | 6.16 | 89.33 | 0.09 | 147.36 | 0.81 | 3.95 | 0.18 | 21.99 | 12.51 |
| | | *0.00* | *0.19* | *4.62* | *0.00* | *9.11* | *0.01* | *0.32* | *0.01* | *2.14* | *1.11* |

## References


Pratscher, J., Dumont, M. G. & Conrad, R. Assimilation of acetate by the putative atmospheric methane oxidizers belonging to the USCα clade. *Environ. Microbiol.* **13,** 2692–2701; 10.1111/j.1462-2920.2011.02537.x (2011).
